# CX3CR1-mediated immune networks in sepsis: implications for precision therapy

**DOI:** 10.1038/s41420-026-03102-1

**Published:** 2026-04-11

**Authors:** Ying Tang, Lu Jia, Yigao Liu, Zhejun Yu, Hui Chen, Ling Liu, Jie Chao, Jianfeng Xie, Haibo Qiu

**Affiliations:** Department of Critical Care Medicine, Jiangsu Provincial Key Laboratory of Critical Care Medicine, Zhongda Hospital, School of Medicine, Southeast University, Nanjing, China

**Keywords:** Infectious diseases, Chemokines

## Abstract

Sepsis is a life-threatening syndrome characterized by profound immune dysregulation in response to infection, and it remains a major cause of mortality worldwide. The CX3C chemokine receptor 1 (CX3CR1) has emerged as a pivotal regulator of sepsis-induced immune dysregulation, orchestrating the proliferation, differentiation, activation, migration, and survival of various immune cell populations, including monocytes/macrophages, natural killer cells (NK), and T cells. Emerging evidence highlights that the diverse expression patterns of CX3CR1 within distinct immune cell subsets determine its dual pro-inflammatory and anti-inflammatory effects, and CX3CR1 expression level is closely correlated with patient survival in sepsis. In addition, targeted modulation of CX3CR1 in specific immune cell types has shown promising efficacy in preclinical sepsis models. This review provides a comprehensive overview of the molecular immunoregulatory networks governed by CX3CR1, its heterogenous functions across different immune subsets, and recent advances in CX3CR1-targeted therapies, highlighting cell type-specific interventions as promising strategies for precision sepsis treatment.

## Facts

- CX3CR1 exerts cell type-specific and even opposing immune functions.
- The role of CX3CL1-CX3CR1 signaling is stage-dependent in sepsis.
- CX3CR1 is associated with sepsis outcomes and has cell-specific prognostic value.
- The CX3CR1-targeted intervention has shown therapeutic potential in sepsis.
- How to modulate CX3CR1⁺ cells without disrupting immune homeostasis remains unresolved.

## Introduction

Each year, sepsis affects an estimated 50 million people worldwide, with more than 20% dying from multiple organ failure driven by immune dysregulation [1, 2]. The pathogenesis of sepsis typically follows a biphasic trajectory of immune imbalance [2, 3]. In the early phase, a hyperinflammatory response—characterized by cytokine storms triggered by pathogen- and damage- associated molecular patterns (PAMPs and DAMPs), leading to systemic inflammation and organ injury. This is followed by a late phase dominated by immunosuppression, with T cell exhaustion and macrophage dysfunction significantly increasing the risk of secondary infections [2, 4]. This dynamic imbalance is jointly driven by dysregulated activation, migration, and cell death across multiple immune cell subsets. Despite decades of research, current clinical management of sepsis still relies primarily on antibiotics, fluid resuscitation, and organ support, with no precise interventions targeting immune dysregulation [2, 5]. Even with standard care, the 28-day mortality rate remains over 20%, and survivors often suffer from long-term immune deficiency and organ dysfunction [6, 7]. Nonspecific immunomodulators, such as corticosteroids, may worsen immune imbalance due to their inability to distinguish between immune cell subsets [8, 9].

Against this backdrop, CX3CR1 has emerged as a promising therapeutic target due to its role as an “immune regulatory hub” within immune cells. CX3CR1 is primarily expressed on immune cells and forms signaling axes with its classical ligand C-X3-C motif chemokine ligand 1 (CX3CL1, also known as fractalkine) and non-classical ligand C-C motif chemokine ligand 26 (CCL26), regulating immune cell migration, activation, effector functions, and fate decisions—functions that are closely associated with sepsis outcomes [10–17]. However, the specific functions of CX3CR1 are highly dependent on cell types.

Notably, CX3CR1 is a G protein-coupled receptor (GPCR) with well-defined structural features and ligand-binding mechanisms (e.g., CRS1/CRS2 binding sites) [18], making it a tractable target for drug development. To date, a few small-molecule inhibitors (such as AZD8797) [19–21] and monoclonal antibodies (such as BI 655088) [22] targeting CX3CR1 have already entered clinical evaluation, supporting its feasibility and translational potential as a therapeutic target. Furthermore, with the continued advancement of in vivo targeted intervention technologies—including adeno-associated virus (AAV)-mediated gene regulation and chimeric antigen receptor T cell (CAR-T) approaches directed toward specific immune cell subsets—it is becoming increasingly feasible to achieve precise modulation of CX3CR1⁺ immune populations, preserving beneficial effects while avoiding harmful immune dysregulation.

Therefore, elucidating the functional heterogeneity of CX3CR1 across immune cell subsets is not only critical for understanding immune imbalance in sepsis but also lays a solid foundation for the development of implementable and translatable personalized precision diagnostic and therapeutic strategies, with significant scientific and clinical implications.

## Structure and signaling mechanisms of CX3CR1

CX3CR1 is a member of the GPCR superfamily and mainly serves as the receptor for CX3CL1, the sole member of the CX3C chemokine subfamily. In addition, CX3CR1 can also interact with CCL26. The seven-transmembrane domain of CX3CR1 enables high-affinity recognition of the CX3C motif, forming the structural basis for its biological functions [10].

CX3CL1 is a specialized chemokine with dual roles as both an adhesion molecule and a chemoattractant [23, 24]. Unlike most chemokines, CX3CL1 is initially produced as a membrane-bound protein that mediates cell adhesion via surface attachment. Inflammatory cytokines upregulate its expression on endothelial cells, promoting leukocyte adhesion and migration into injured tissues [23, 24]. Under pathological conditions, CX3CL1 is cleaved by a disintegrin and metalloproteinase 10/17 (ADAM10/17) into a soluble form, which binds CX3CR1, internalizes into cells, and activates chemotactic signaling pathways [23–25]. Elevated serum levels of soluble CX3CL1 correlate with increased proinflammatory cytokine [26]. These features enable CX3CL1 to function effectively in both membrane-anchored and soluble states [27].

The structural conformation of CX3CR1 is closely linked to its functional state [25]. At rest, CX3CR1 exists as a monomer. Binding of CX3CL1, which possesses a distinct α-helical structure and disulfide-linked domains, engages the N-terminal region of CX3CR1 in a wedge-like manner, triggering conformational changes that promote receptor dimerization/oligomerization and subsequent activation [18]. This facilitates the exchange of guanosine diphosphate (GDP) for guanosine triphosphate (GTP) on the associated Gα subunit, initiating downstream signaling cascades [23, 24, 28]. CX3CL1 binds CX3CR1 through a two-site model [18, 29]: its globular domain contacts the extracellular chemokine recognition site 1 (CRS1), while its N-terminal segment penetrates deeply into the transmembrane helical bundle at chemokine recognition site 2 (CRS2), establishing extensive polar and hydrophobic interactions. Key residues within CX3CR1, such as E254⁶.⁵⁸ and E279⁷.³⁹, form critical salt bridges and polar contacts with CX3CL1, as indicated by mutagenesis and functional assays [18]. Structural studies reveal that the short β-hairpin of CX3CR1’s extracellular loop 2 uniquely accommodates the 30s-loop of CX3CL1, preventing steric clashes observed in other chemokine receptors and thereby contributing to the receptor’s high ligand specificity [18].

CX3CR1’s unique structural and signaling characteristics—particularly its specific ligand binding—underscore its central role in both physiological regulation and disease pathogenesis [30]. Inflammatory stimuli such as lipopolysaccharide (LPS), interleukin-1 beta (IL-1β), interleukin-8 (IL-8), tumor necrosis factor alpha (TNF-α), and interferon gamma (IFN-γ) enhance the expression of CX3CL1 [13, 31, 32]. The interaction of CX3CR1-CX3CL1 sets off multiple downstream signaling networks, including FAS/FASL [33], PI3K/Akt [13, 34], MAPK/ERK [35], NF-κB [13], Src/FAK [36], CCR1/CCR5 [11], JAK/STAT [37], GZMB/perforin [38] and IFN-γ [39], which orchestrate a range of cellular functions such as migration, cell death, angiogenesis, proliferation, polarization, inflammatory response, and host defense against pathogens [11, 13, 19, 33, 37–43].

**Fig. 1:**
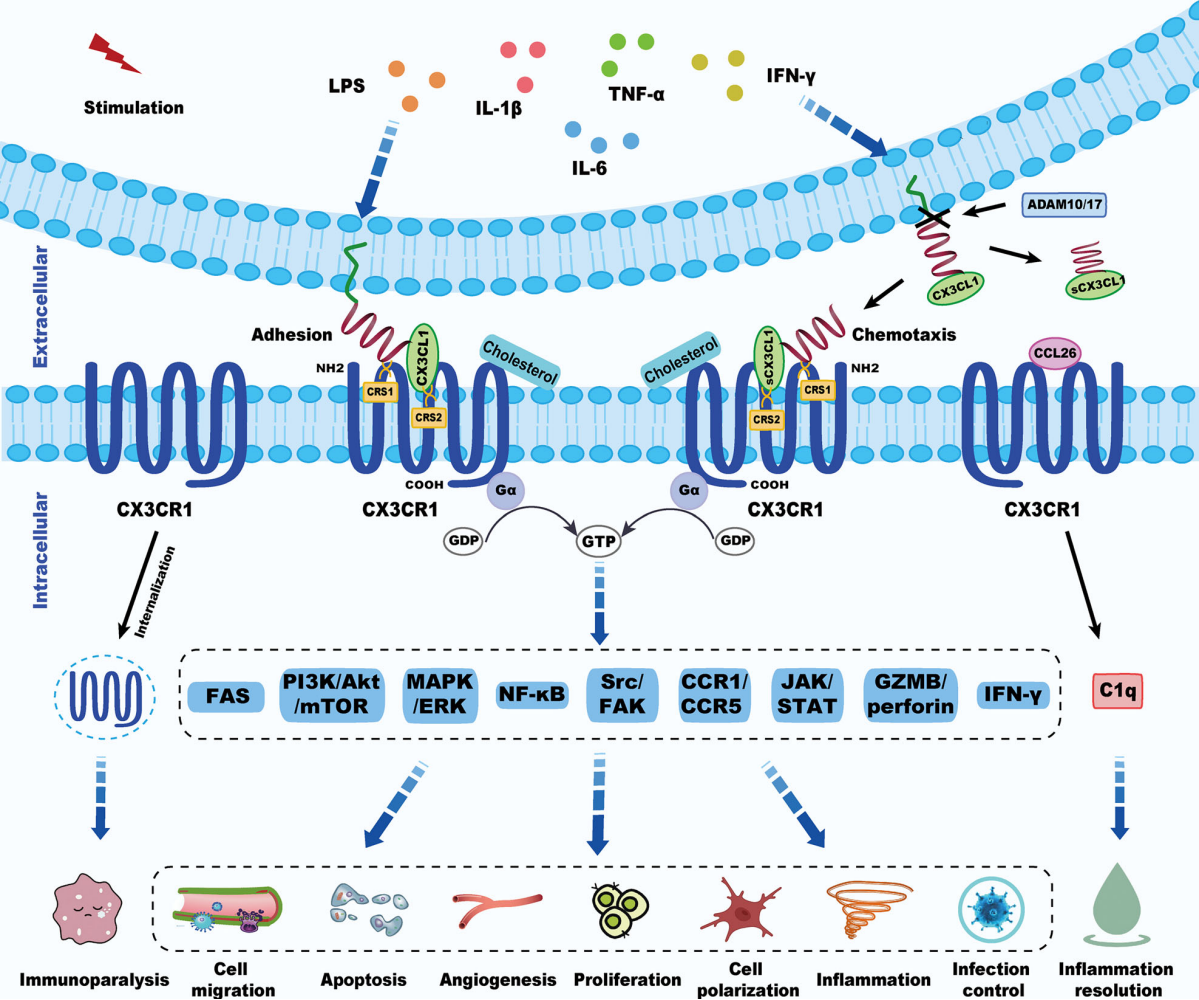
The structure, function, and signaling pathways of CX3CR1. Under inflammatory stress, CX3CL1 expression is upregulated in response to upstream signals such as LPS, IL-1β, IL-8, TNF-α, and IFN-γ. Membrane-bound CX3CL1 promotes leukocyte adhesion and can be cleaved by ADAM10/17 to generate soluble CX3CL1, which binds to CX3CR1 and acts as a chemotactic factor. Through two specific contact sites, CRS1 and CRS2, CX3CL1 engages CX3CR1 and, in the presence of cholesterol, triggers G protein activation, initiating downstream cascades including FAS/FASL, PI3K/Akt/mTOR, MAPK/ERK, NF-κB, and JAK/STAT pathways. These pathways collectively regulate cellular processes such as migration, apoptosis, angiogenesis, proliferation, polarization, inflammation, and infection control. CX3CR1 internalization from the cell membrane may contribute to immunoparalysis. In addition, CX3CR1 binding to the non-classical ligand CCL26 has been shown to promote resolution of inflammation.

For instance, in the peripheral blood of patients with lung cancer, CX3CR1⁺ T cells exhibit higher levels of FAS/FASL pathway-mediated apoptosis [33]. Under conditions of chronic inflammation, activation of the CX3CL1-CX3CR1 axis stimulates the PI3K/Akt signaling pathway, reduces the production of IL-1β, NO (nitric oxide), IL-6, and TNF-α, exerts anti-inflammatory effects, suppresses LPS-induced microglial polarization, and decreases neuronal death [13, 34]. In addition, CX3CR1 may promote monocyte survival through PI3K/Akt signaling [44]. In the hippocampus of depressive mice, the CX3CL1-CX3CR1 axis exacerbates inflammatory responses by upregulating MAPK14 phosphorylation [35]. CX3CL1 can also enhance renal inflammation and fibrosis by promoting MAPK phosphorylation and facilitating NF-κB translocation from the cytoplasm to the nucleus [13]. In renal cell carcinoma, tumor cell migration and invasion depend on CX3CR1-mediated activation of Src/FAK signaling [36]. However, following hepatic ischemia-reperfusion injury, CX3CR1 deficiency in macrophages paradoxically induces compensatory upregulation of other migration-related receptors, such as CCR1 and CCR5, thereby promoting macrophage migration [11]. Moreover, the CX3CL1-CX3CR1 axis can drive macrophage polarization toward an M2 phenotype via the JAK2/STAT3 pathway, leading to increased secretion of anti-inflammatory cytokines [37]. In patients with systemic lupus erythematosus, circulating CX3CR1⁺CD8⁺ T cells exhibit elevated expression of perforin and granzyme B (GZMB), enhancing their cytotoxic and antimicrobial capacity [38]. During acute Toxoplasma infection, caspase-1 expression is upregulated in CX3CR1⁺ cells, promoting interleukin-18 (IL-18) release, which in turn enhances IFN-γ production by CD4⁺ T cells and strengthens parasite control [39]. These varied downstream pathways highlighting its potential as a promising therapeutic target (Fig. 1).

## Stage-dependent roles of CX3CR1-mediated signaling in sepsis

Sepsis is a dynamic syndrome that typically progresses from an early hyperinflammatory state to a later immunosuppressive phase. CX3CR1-mediated signaling exerts stage-dependent effects during sepsis progression; for example, in the early phase, CX3CR1 promotes inflammatory responses through activation of pathways such as MAPK/ERK, whereas in the later phase, it supports immune cell survival via pathways including PI3K/Akt/mTOR [24, 45–47].

During the early phase of sepsis, the CX3CL1-CX3CR1 axis plays a critical role in host defense against bacterial infection by enhancing the bactericidal activity of phagocytes, promoting inducible nitric oxide synthase (iNOS)-dependent NO production and proinflammatory cytokine release via NF-κB activation [48], and augmenting interferon responses in NK and T cells [38, 39]. At the same time, this axis facilitates immune cell recruitment and adhesion, thereby amplifying inflammatory responses via increased production of pro-inflammatory mediators [49]. Moreover, CX3CL1-CX3CR1 signaling has been shown to activate the JAK/STAT signaling pathway, further exacerbating inflammation in rat models of severe acute pancreatitis [50]. Under LPS challenge, activation of the PCSK9/HMGB1/NF-κB/NLRP3 and CX3CL1/CX3CR1 pathways is associated with increased reactive oxygen species (ROS) production and suppression of the Nrf2/HO-1 antioxidant axis, leading to enhanced NF-κB signaling. Pharmacological inhibition of PCSK9 with alirocumab disrupts the feed-forward loop between NF-κB/TNF-α and CX3CL1/CX3CR1 signaling, thereby attenuating inflammatory cascades and markedly reducing LPS-induced inflammation [51]. Interestingly, CX3CR1 contributes to the synthesis and release of anti-inflammatory mediators, including IL-1 receptor antagonist (IL-1Ra) and prostaglandin E2 [49].

**Fig. 2:**
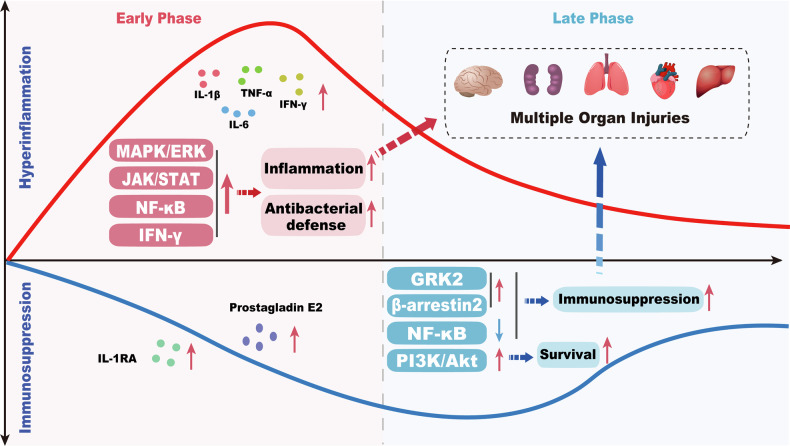
Dynamic changes of the CX3CR1-mediated signaling in sepsis. Sepsis progresses from an early hyperinflammatory-dominant phase to a later immunosuppressive-dominant phase, during which CX3CR1 signaling exhibits stage-dependent dynamics. In the early phase, the CX3CL1-CX3CR1 axis activates MAPK/ERK, NF-κB, and JAK/STAT pathways, enhancing phagocytic bactericidal activity, proinflammatory cytokine release, and immune cell recruitment, thereby amplifying inflammatory cascades; meanwhile, it also promotes the production of anti-inflammatory mediators such as IL-1Ra and prostaglandin E2. In the late phase, GRK2/β-arrestin2-mediated CX3CR1 internalization is enhanced, leading to reduced surface expression and diminished immune cell responsiveness. CX3CR1 signaling downregulates NF-κB activity at this stage, contributing to the development of immunosuppression; however, it concurrently supports the survival of certain immune cell subsets via pathways such as PI3K/Akt.

In the late phase of sepsis, upregulation of the CX3CR1-CX3CL1 axis has been correlated with organ dysfunction such as acute kidney injury in this phase [52]. During this immunoparalysis phase, surface expression of CX3CR1 on monocytes/macrophages is markedly reduced, primarily due to enhanced receptor internalization that weakens CX3CL1-mediated immune responses [53, 54], rather than merely transcriptional suppression as previously described. Raspé et al. reported that in this phase, CX3CL1 expression is upregulated whereas CX3CR1 expression is downregulated, which might be regulated by NF-κB probably via attenuated release of inflammatory cytokines [55]. In addition, previous studies have shown that CX3CR1 signaling promotes the survival of certain key immune cell subsets during the late phase of sepsis [44, 45, 56] (Fig. 2).

## The potential value of CX3CR1 as a clinical biomarker in sepsis

Several studies have reported an association between higher CX3CR1 expression and better clinical outcomes in sepsis. CX3CR1 was identified as one of the most significantly highly differentially expressed genes distinguishing survivors from non-survivors in two independent septic shock cohorts, shows strong diagnostic potential (AUC = 0.982) [57]. The cyclin-dependent kinase 1 (CDK1)/CX3CR1 ratio has been proposed as a survival-associated biomarker that discriminates septic patients into three groups based on its level, with intensive care unit mortality increasing stepwise as the ratio rises [58]. A prospective, multicentre, non-interventional study indicated that decreased CX3CR1 mRNA expression was independently associated with increased short- and long-term mortality in critically ill patients, and suggested that reduced CX3CR1 expression in septic patients may represent an additional feature of sepsis-induced immunosuppression [59]. Another study identified CX3CR1-related molecular signatures that were associated with the diagnosis and prognosis of sepsis and ARDS [60].

In addition to established sepsis immunosuppression biomarkers such as HLA‑DR and programmed death-1 (PD-1)/programmed death ligand-1 (PD-L1), emerging evidence suggests that CX3CR1 may provide complementary prognostic information. Decreased HLA-DR expression primarily reflects impaired antigen presentation, whereas upregulation of PD‑1/PD‑L1 indicates T-cell exhaustion during sepsis-associated immunosuppression [61, 62]. In contrast, CX3CR1 is broadly expressed across multiple immune cell types [63] and exhibits more diverse functional implications. Notably, its prognostic relevance appears to be highly cell type-dependent. An increased proportion of peripheral CX3CR1⁺ intermediate monocytes (CD14⁺CD16⁺) positively correlates with 28-day survival in patients with sepsis [64]. Conversely, CX3CR1 expression on CD4⁺ T cells has been linked to poor prognosis [65]. These findings suggest that CX3CR1 may exert opposing effects across distinct immune cell populations, underscoring the importance of systematically delineating its cell-specific functions and prognostic associations. Such efforts will further strengthen its potential as both a clinical biomarker and a therapeutic target in sepsis.

## CX3CR1 functional diversity across immune subsets in sepsis

CX3CR1 is widely expressed on immune cells such as monocytes, macrophages, NK cells, and T cells, and plays a central role in sepsis by integrating inflammatory cues and regulating immune cell fate through diverse signaling programs. Crucially, the effects of CX3CR1 signaling are not uniform across the immune system. Rather, its function varies dramatically between immune subsets, shaped by cell-intrinsic signaling programs and local microenvironmental factors. For instance, CX3CR1 promotes monocyte/macrophage survival, thereby contributing to the resolution of inflammation [34], yet also enhances interferon responses in NK and T cells to control infection [38, 39, 43]. In contrast, CX3CL1-CX3CR1 axis amplifies and sustains T helper 1 cells (Th1)-driven inflammation [32], while toll-like receptor 4 (TLR4)-induced G protein-coupled receptor kinase 2 (GRK2) and β-arrestin2 promote CX3CR1 internalization, suppress NF-κB activation, and dampen inflammation, contributing to sepsis-induced immunoparalysis [54]. These findings underscore the subset-specific roles of CX3CR1 in orchestrating immune activation and resolution during sepsis. A deeper understanding of its diverse functions across immune cell types will be essential for developing targeted immunotherapies aimed at restoring immune balance in sepsis.

### Role of CX3CR1 in monocytes and macrophages

Monocytes and their derivatives, macrophages, are key immune cells that maintain homeostasis and mediate inflammatory responses. CX3CR1 plays multifaceted roles in inflammation, fibrosis, cancer, and metabolic disorders by regulating the development, migration, activation, and fate of monocytes and macrophages [66–68].

#### Role of CX3CR1 signaling in monocyte/macrophage differentiation, survival and death

CX3CR1 signaling plays a critical role in the differentiation of hematopoietic stem and progenitor cells (HSPCs), promoting the generation of microglia, a specialized macrophage population in the brain. Targeting the CX3CR1 locus enables epigenetically regulated transgene expression—silenced in stem-like cells and activated in differentiated myeloid cells [56]. Consistently, CX3CR1⁺/⁻ HSPCs exhibit enhanced engraftment in the central nervous system (CNS) and preferentially generate microglia-like progeny, highlighting the therapeutic potential of CX3CR1 in neuroregeneration and lineage-directed therapies [69]. Moreover, CX3CR1 deficiency also reduces the survival of dendritic cells (DCs), monocytes, and macrophages compared to their CX3CR1-expressing counterparts [67]. Notably, a distinct population of resident arterial macrophages resides within the arterial wall. These cells originate from CX3CR1⁺ embryonic precursors and early postnatal bone marrow-derived monocytes. In adulthood, their maintenance relies mainly on local proliferation (rather than monocyte recruitment), including after sepsis-induced depletion, and their survival within the vascular niche is critically dependent on the CX3CR1-CX3CL1 axis [70]. In human monocytes, CX3CL1 effectively rescues serum starvation-induced cell death; however, this protective effect is completely abolished following CX3CR1 mutation. In a mouse model of Candida infection, CX3CR1 deficiency results in reduced macrophage survival, impaired fungal clearance in tissues, and increased mortality [71]. These findings establish CX3CR1 as a key regulator of monocyte/macrophage differentiation, survival and death.

#### Role of CX3CR1 signaling in monocyte/macrophage polarization and migration

CX3CL1/CX3CR1 signaling promotes microglial activation and migration to aid the repopulation of microglia following ablation [72]. In nonalcoholic steatohepatitis, elevated CX3CR1 in M1 liver macrophages restrains disease progression by limiting monocyte infiltration and M1 polarization, whereas CX3CR1 deficiency aggravates inflammation and fibrosis [68]. In intervertebral disc degeneration, the CX3CL1-CX3CR1 axis drives M2-like macrophage polarization and suppresses inflammation [37]. In primary Sjögren syndrome, the CX3CL1-CX3CR1 axis regulates macrophage function predominantly by promoting M2-like polarization, thereby contributing to anti-inflammatory responses [73]. Moreover, research on CX3CR1 gene polymorphisms found that the I249 allele is associated with enhanced monocyte adhesion and reduced kidney injury [74]. However, CX3CR1-mediated monocyte/macrophage activation and migration may also drive pathology. CX3CL1/CX3CR1 signaling contributes to neuroglial activation and photoreceptor degeneration in retinal degeneration [21]. In hypoxic postconditioning, CX3CR1 is upregulated in neurons but downregulated in glial cells, reducing microglial activation and neuronal protection in the hippocampal CA1 region [75]. In liver fibrosis, splenic CX3CR1⁺ monocytes migrate to fibrotic tissue and secrete pro-fibrotic mediators [76]. Interestingly, CX3CR1 deficiency or inhibition may facilitate inflammation resolution by enhancing macrophage migration via CCR1/CCR5 pathway [11].

In sepsis, CX3CR1⁺ monocytes are recruited to inflamed tissues such as the lungs, kidneys, and liver. In sepsis-induced lung injury, CX3CR1^+^ macrophages were recruited into the lungs and considered as valuable diagnostic tools for postmortem diagnosis [77]. Some studies report that CX3CL1-CX3CR1 signaling drives monocyte recruitment and proinflammatory activation [12], contributing to tissue injury and fibrosis [78, 79]. In contrast, in murine cecal ligation and puncture (CLP)-induced sepsis, CX3CR1 facilitates early monocyte recruitment to the kidney and limits tissue damage through IL-1ra secretion by Ly6C^high^ monocytes, transferring CX3CR1-competent monocyte-rich bone marrow cells into CX3CR1-deficient septic mice significantly reduced kidney injury and improved survival [74, 80].

Thus, CX3CR1 directs monocyte/macrophage polarization and migration in a context-dependent manner, shaping either tissue injury or protection.

#### Role of CX3CR1 signaling in monocyte/macrophage metabolic reprogramming

CX3CR1 signaling plays a multifaceted role in shaping monocyte/macrophage metabolic reprogramming across inflammatory and tissue-specific contexts. In visceral adipose tissue, CX3CR1^hi^ macrophages activate the Arg1-polyamine axis to enhance eukaryotic initiation factor 5 A hypusination and mitochondrial function, thereby promoting oxidative metabolism and alleviating adipose-derived stem cells senescence [81]. In tumor and chronic inflammatory microenvironments, CX3CR1⁺ macrophages exhibit distinct metabolic and functional phenotypes associated with cytokine production and immune modulation [82]. Activation of the CX3CL1/CX3CR1 axis also shifts cellular metabolism from proinflammatory glycolysis toward oxidative phosphorylation, facilitating inflammatory resolution [83]. Furthermore, CX3CR1 signaling integrates inflammatory and iron metabolic pathways by regulating proinflammatory cytokines, hepcidin expression, and iron storage and utilization in macrophages, thereby influencing antimicrobial activity and redox homeostasis [84]. Collectively, CX3CR1 serves as a central immunometabolic regulator in monocytes and macrophages.

#### Role of CX3CR1 signaling in monocyte/macrophage-mediated tissue repair

In Alzheimer’s disease, CX3CR1 deficiency impairs microglial clearance of Aβ and plaque interaction, leading to a degenerative microglial phenotype with dysregulated inflammation, underscoring CX3CR1’s neuroprotective role [85]. In cerebral small vessel disease, transient infiltration of CX3CR1⁺ monocytes into the hippocampus facilitates neurovascular repair at microinfarct sites [86]. CX_3_CR1^high^ Ly6C^−^ monocyte subset persists longer in tissues and serves as a precursor for resident myeloid cells in noninflamed tissues including liver, lung, brain, and spleen [87]. Additionally, depletion of CX3CR1⁺ macrophages has been shown to exacerbate sepsis-associated kidney injury [88]. These findings underscore the reparative potential of CX3CR1⁺ myeloid cells.

#### Role of CX3CR1 signaling in monocyte/macrophage-mediated antibacterial defense and inflammation

CX3CR1 modulates monocyte/macrophage function through intricate signaling mechanisms in inflammatory environments. CX3CL1 enhances IL-1β, TNF-α, IFN-γ, and interleukin-12 (IL-12) expression and activates NF-κB signaling in macrophages. The CX3CL1/CX3CR1 axis is essential for antibacterial defense by enhancing iNOS-mediated NO production, inducing proinflammatory cytokine release via NF-κB activation, and promoting phagocyte bactericidal activity [36]. In allergic lung inflammation, a CX3CR1⁺ alveolar macrophage subset expands after allergen exposure; CCL26 secreted by epithelial cells stimulates these macrophages to produce complement component 1q (C1q), aiding in the resolution of eosinophilic inflammation [14].

In sepsis, CD16⁺ monocytes warrant particular attention. They markedly expand in the peripheral blood of septic patients, express high levels of CX3CR1 and undergo efficient transendothelial migration in the presence of CX3CL1 [63]. Moreover, during their interaction with endothelial cells, CD16⁺CX3CR1⁺ monocytes produce substantial amounts of IL-6, C-C motif chemokine ligand 2, and matrix metalloproteinase-9 [89]. In addition, LPS enhances TNF-α secretion by CD16⁺ monocytes; TNF subsequently induces endothelial cells to release soluble CX3CL1, which downregulates surface CX3CR1 expression on CD16⁺ monocytes, reduces their adhesion capacity, and suppresses TNF-α release through a negative feedback mechanism [90]. Additionally, Finsterbusch et al. show that in sepsis-induced acute kidney injury, CX3CR1^high^ monocytes establish direct cell-cell contacts with neutrophils and produce proinflammatory cytokines, such as TNF-α, to activate the release of ROS from neutrophils [91].

Overall, CX3CR1 orchestrates monocyte/macrophage-mediated antimicrobial activity and inflammatory responses.

#### Role of CX3CR1 signaling in monocyte/macrophage-mediated immunosuppression

Within the tumor microenvironment, CX3CR1⁺ monocyte-derived macrophages contribute to immune evasion by producing immunosuppressive factors [28]. *Fusobacterium nucleatum* induces CX3CR1 expression in tumor-infiltrating neutrophils and macrophages, forming a CX3CR1⁺PD-L1(programmed death ligand-1)⁺ population that transfers intracellular bacteria to tumor cells and establishes an immunosuppressive niche [92]. Additionally, CX3CR1⁺CD206⁺ macrophages accumulate during tumor progression, contributing to the formation of an immunosuppressive myeloid compartment [93]. In sepsis, CX3CR1 signaling may impair bacterial clearance by inhibiting phagocytosis of macrophage and neutrophils [31]; meanwhile, internalization of CX3CR1 in macrophages may facilitate the establishment of an immunosuppressive microenvironment [54]. However, other studies have reported that during septic shock, CX3CR1 expression is markedly downregulated—particularly in non-survivors—thereby impairing monocyte responsiveness to CX3CL1 and potentially contributing to immune paralysis [53]. These data highlight the context-dependent role of CX3CR1 in monocyte/macrophage-driven immunosuppression.

Together, these findings highlight the context-dependent, dual roles of CX3CR1 in monocytes and macrophages—where it may either exacerbate or resolve inflammation depending on cell subtype and microenvironmental cues. Dysregulation of this pathway represents a potential mechanism underlying sepsis-induced immune dysfunction.

### Role of CX3CR1 in natural killer cells

NK cells are innate lymphocytes that serve as first-line defenders, rapidly eliminating infected or transformed cells and shaping subsequent immune responses. CX3CR1 enhances the chemotactic migration and tissue homing capacity of NK cells via cooperation with C-X-C motif chemokine receptor 1 (CXCR1) and C-X-C motif chemokine receptor 2 (CXCR2), thereby modulating immune microenvironments across various tissues [94]. Within the tumor microenvironment, downregulation of CX3CR1 correlates with impaired NK cell activity [95]. The CX3CL1-CX3CR1 axis not only facilitates NK cell-mediated tumor cell clearance [96] but can also be exploited by tumors to recruit myeloid-derived suppressor cells (MDSCs) and other immunosuppressive populations, thereby promoting immune evasion [28, 97]. In melanoma, NK cells are recruited to the tumor regions via CX3CR1, where they restrict CD8⁺ T cell infiltration and establish immune-excluded tumor regions, ultimately diminishing the efficacy of immune checkpoint blockade (ICB); Depleting NK cells in mouse models reversed immune exclusion and enhanced tumor clearance, highlighting a CX3CR1-dependent role of NK cells in limiting ICB response [98]. In pediatric acute myeloid leukemia, high CX3CR1 expression is associated with poor prognosis, suggesting a potential role in suppressing antitumor immunity [99]. Furthermore, in a mouse model of schistosomiasis-induced liver fibrosis, single-cell sequencing identified an expansion of the CX3CR1⁺ NK cell subset at week four post-infection, suggesting a dynamic role for this subset during fibrosis progression [100]. These multifaceted roles of CX3CR1 in regulating NK cell migration and immune function indicate its potential involvement in immune surveillance or dysregulation during sepsis.

### Role of CX3CR1 in T cells

The role of CX3CR1 in innate immune cells, such as monocytes and macrophages, was established early on, but its important functions in adaptive immunity have only been increasingly recognized in recent years. As central players in the adaptive immune system, T cells are crucial for immune surveillance, pathogen clearance, and long-term immune memory. Recent studies increasingly recognize CX3CR1 as a critical marker of functional heterogeneity among T cell subsets [101]. Zwijnenburg et al. suggested that graded expression of CX3CR1 delineates the differentiation continuum of both CD4⁺ and CD8⁺ T cells in humans and mice. CX3CR1 levels, combined with CD62L, distinguish functionally and migratorily distinct T cell subsets and enable cross-species comparison [102].

CX3CR1⁺ exert functions in CD4⁺ T cells in multiple diseases. CX3CR1 is predominantly expressed by proinflammatory Th1 and cytotoxic immune cells, and its engagement with CX3CL1 supports sustained Th1 proinflammatory responses [32]. Tran et al. suggested that BCG-induced CX3CR1^hi^ CD4⁺ memory T cells accumulate in the lungs and circulation, exerting early and antigen-independent antiviral protection through IFN-γ and enhancing alveolar macrophage antimicrobial activity [103]. Akiyama et al. identified a CX3CR1⁺ cytotoxic CD4⁺ T cell subset enriched in untreated late-onset rheumatoid arthritis, characterized by granzyme B expression and ThA-like features, which correlated with disease activity after anti-inflammatory therapy, whereas CX3CR1⁺CD8⁺ T cells showed age-related but not disease-relate increases [104]. CX3CR1⁺ CD4⁺ T cells with cytotoxic properties have also been observed in primary Sjögren’s syndrome [43], and they exhibit tissue homing and survival in parasitic infection [105].

CX3CR1 is stably expressed in memory CD8⁺ T cells but is difficult to induce in naïve T cells [102]. Based on CX3CR1 expression, CD8⁺ memory T cells can be classified into three subsets: CX3CR1^lo^ central memory T cells with high self-renewal and lymphoid tissue homing capacity; CX3CR1^int^ peripheral memory T cells surveilling non-lymphoid tissues; and CX3CR1^hi^ circulating effector memory T cells with enhanced cytotoxicity and migratory potential [106]. CX3CR1⁺CD8⁺ T cells exhibit strong cytotoxicity, characterized by high expression of perforin and granzyme B [38, 43]. However, a study reported that these cells show a selective survival disadvantage, which is exacerbated by necrotic cell-derived factors, and this disadvantage cannot be rescued by CX3CL1 stimulation [40]. In contrast, other studies have shown that CX3CR1⁺CD8⁺ T cells possess enhanced antitumor activity, greater effector cytokine production, and superior mitochondrial function. By comparison, CX3CR1⁻CD8⁺ T cells are more prone to differentiate toward an exhausted phenotype and exhibit a stronger tendency toward terminal differentiation following adoptive transfer, suggesting that CX3CR1 may delay T cell exhaustion by preserving mitochondrial function [107].

CX3CR1 also plays a pivotal role in CD8⁺ T cells across a range of diseases. A distinct CX3CR1⁺ effector-like exhausted CD8⁺ T cell subset (Tex_eff-like_) that retains cytolytic activity and expands in response to immune checkpoint blockade, correlating with better clinical outcomes. Although their lineage trajectory from progenitor exhausted T cells (Tex) remains unclear, their unique epigenetic profile makes them attractive therapeutic targets in infection and cancer [108]. In tumors, CX3CR1⁺CD8⁺ T cells migrate to tumor sites via the CX3CL1-CX3CR1 axis and exert antitumor activity [28], with their abundance positively correlated with anti-PD-1 response and overall patient survival [33, 109]. In lung cancer, circulating CX3CR1⁺CD8⁺ T cells possess potent effector functions and tumor-reactivity; their adoptive transfer, especially combined with PD-1 blockade, enhances anti-tumor efficacy and improves survival, highlighting their potential for personalized immunotherapy [33].

In sepsis, CX3CR1⁺ T cells play dual roles. CX3CR1⁺CD8⁺ tissue-resident memory T cells accumulate in the brain during sepsis-associated encephalopathy, contributing to neuroinflammation [110], whereas CX3CR1⁺ regulatory T cells (Tregs) alleviate neuroinflammation by modulating microglial polarization [111].

Overall, CX3CR1 governs T cells development, differentiation, migration, effector function, and fate determination, suggesting a pivotal yet incompletely defined role in sepsis pathophysiology that warrants further investigation.

### Role of CX3CR1 in other immune cell types

**Fig. 3:**
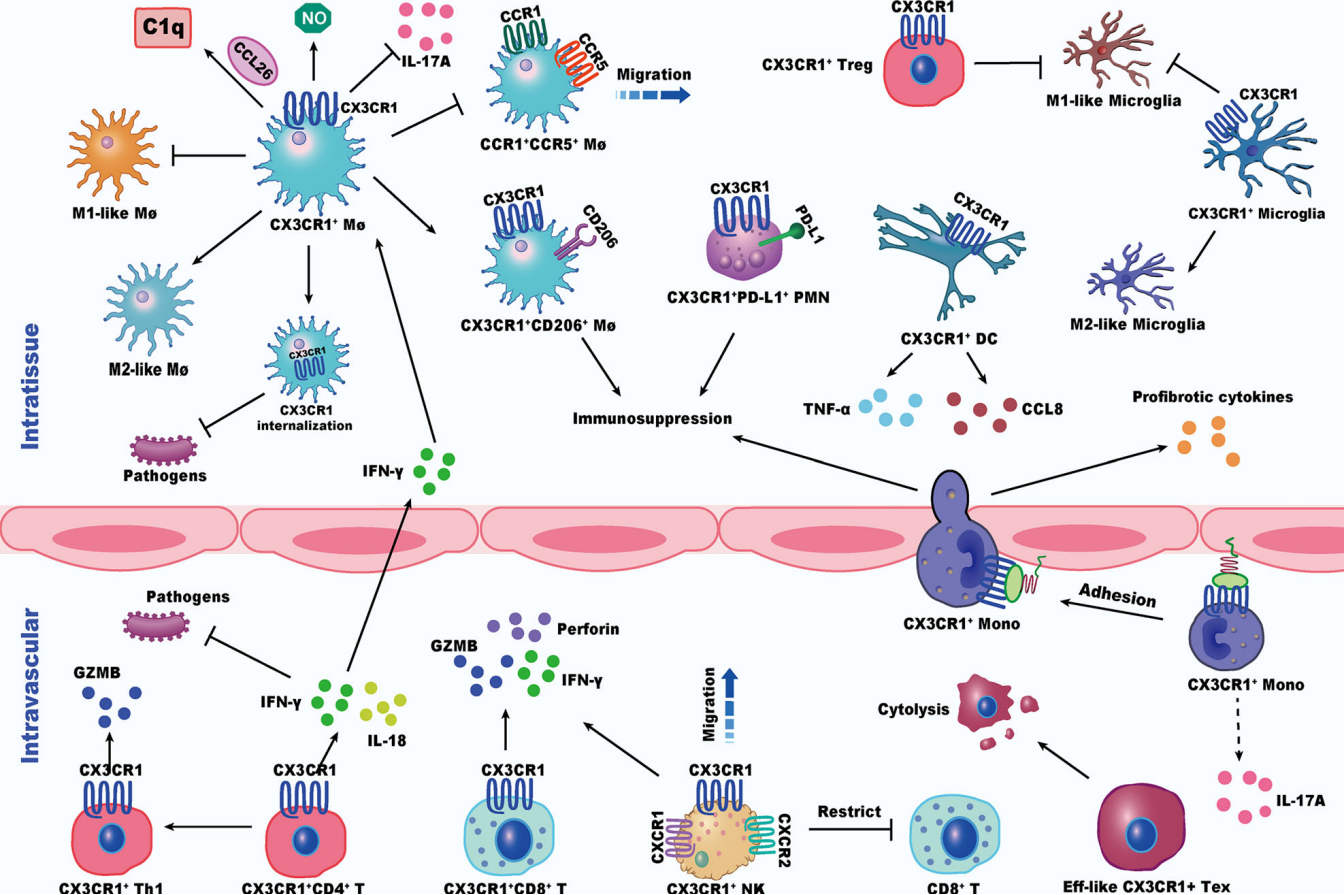
CX3CR1 functional diversity across immune subsets implicated in sepsis. In tissues, CX3CR1⁺ macrophages bind CCL26 and secrete C1q to promote eosinophil clearance, while also producing IL-17A to suppress Th17 responses. In sepsis, increased CX3CR1 internalization impairs pathogen defense. Upregulated CX3CR1 promotes M1 polarization, whereas inhibition of the CX3CR1-CX3CL1 axis facilitates M2 polarization, enhances CCR1/CCR5 expression in hepatic macrophages, and promotes CX3CR1⁺CD206⁺ macrophage differentiation. CX3CR1⁺ PMNs exert immunosuppressive effects via PD-L1. CX3CR1⁺ DCs release proinflammatory cytokines (e.g., CCL8, TNF-α), and in the brain, CX3CR1⁺ microglia collaborate with Tregs to inhibit M1-like microglia and reduce neuroinflammation. In the vasculature, proinflammatory signals upregulate CX3CR1 on Th1 but not T helper 2 cells (Th2) cells. CX3CR1⁺ Th1 cells express GZMB and are cytotoxic; CX3CR1⁺ CD4⁺ T cells produce IFN-γ and IL-18 to enhance anti-pathogen responses. CX3CR1⁺ CD8⁺ T and NK cells are cytotoxic, with CD8⁺ T cells releasing IFN-γ, perforin, and GZMB, while NK cells are migratory and may suppress CD8 function. CX3CR1 also marks early exhausted effector-like T cells. CX3CR1⁺ monocytes exhibit strong trans-endothelial migration and adhesion, secreting IL-17A and profibrotic cytokines upon tissue infiltration. MΦ: macrophages.

Beyond monocytes/macrophages, NK cells, and T cells, CX3CR1 expressed on other immune cell types also contributes to the shaping of the immune microenvironment and may influence sepsis progression. CX3CR1⁺ DCs mediate the onset and progression of non-infectious uveitis by secreting pro-inflammatory cytokines such as TNF-α [112], and in aging tissues such as the epididymis, they promote immune cell infiltration and cytokine release, exacerbating local inflammation [113]. Mohapatra et al. found that CX3CR1 deficiency, whether alone or combined with C-C chemokine receptor type 2 (CCR2) deficiency, increased bacterial loads in the mediastinal lymph nodes and disrupted the localization of Ly6C^lo^ monocyte-derived DCs [114]. In sepsis, DC depletion and dysfunction are key drivers of immunosuppression [115]. Additionally, CX3CR1 and CXCR2 signaling coordinate the exercise-induced recruitment of intramuscular neutrophils, supporting muscle immune homeostasis under inflammatory conditions [116]. However, the roles of CX3CR1 across diverse immune cell types in sepsis remain poorly understood and require further investigation (Fig. 3).

## Potential cell-type-based therapy targeting CX3CR1 in sepsis

CX3CR1 plays dual roles in the immunoregulation of sepsis. Based on current evidence, therapeutic strategies targeting CX3CR1 in sepsis can be developed along multiple dimensions.

### Potential strategies targeting monocytes and macrophages

The regulatory strategies of CX3CR1 in the monocyte-macrophage system remains controversial, potentially exhibiting spatiotemporal specificity and depending on the immune activation state. We can achieve the upregulation or downregulation of CX3CR1 in monocytes and macrophages through various approaches, including pharmacological interventions, gene therapies, and cell-based treatments.

In terms of pharmacological interventions, the selective CX3CR1 antagonist JMS-17-2 has been shown to reduce pro-inflammatory cytokine production in monocytes by inhibiting p38/JNK phosphorylation [35, 117], while AZD8797, another CX3CR1 inhibitor, disrupts the CX3CL1-CX3CR1 axis and suppresses monocyte recruitment to sites of inflammation [118]. In the future, approaches such as lipid nanoparticle (LNP)-mediated delivery could be employed to target these small-molecule compounds to the vicinity of specific cells.

With regard to gene therapies, CRISPR-Cas9-mediated knockout of CX3CR1 can enhance the phagocytic activity of macrophages derived from human induced pluripotent stem cells, although this may also intensify inflammatory responses [119]. A recent study showed that shRNA-mediated silencing of TNF-α and IGF1 in CX3CR1⁺ macrophages via lentiviral transduction in bone marrow cells effectively suppressed thoracic aortic aneurysm development [120], suggesting that similar intervention strategies could be explored in the context of sepsis. In humans, the I249 CX3CR1 allele, known to increase receptor adhesion, correlated with reduced risk of sepsis-induced acute kidney injury, suggesting that gene editing to induce the expression of the I249 variant may offer a potential strategy for personalized therapy in sepsis [74].

**Fig. 4:**
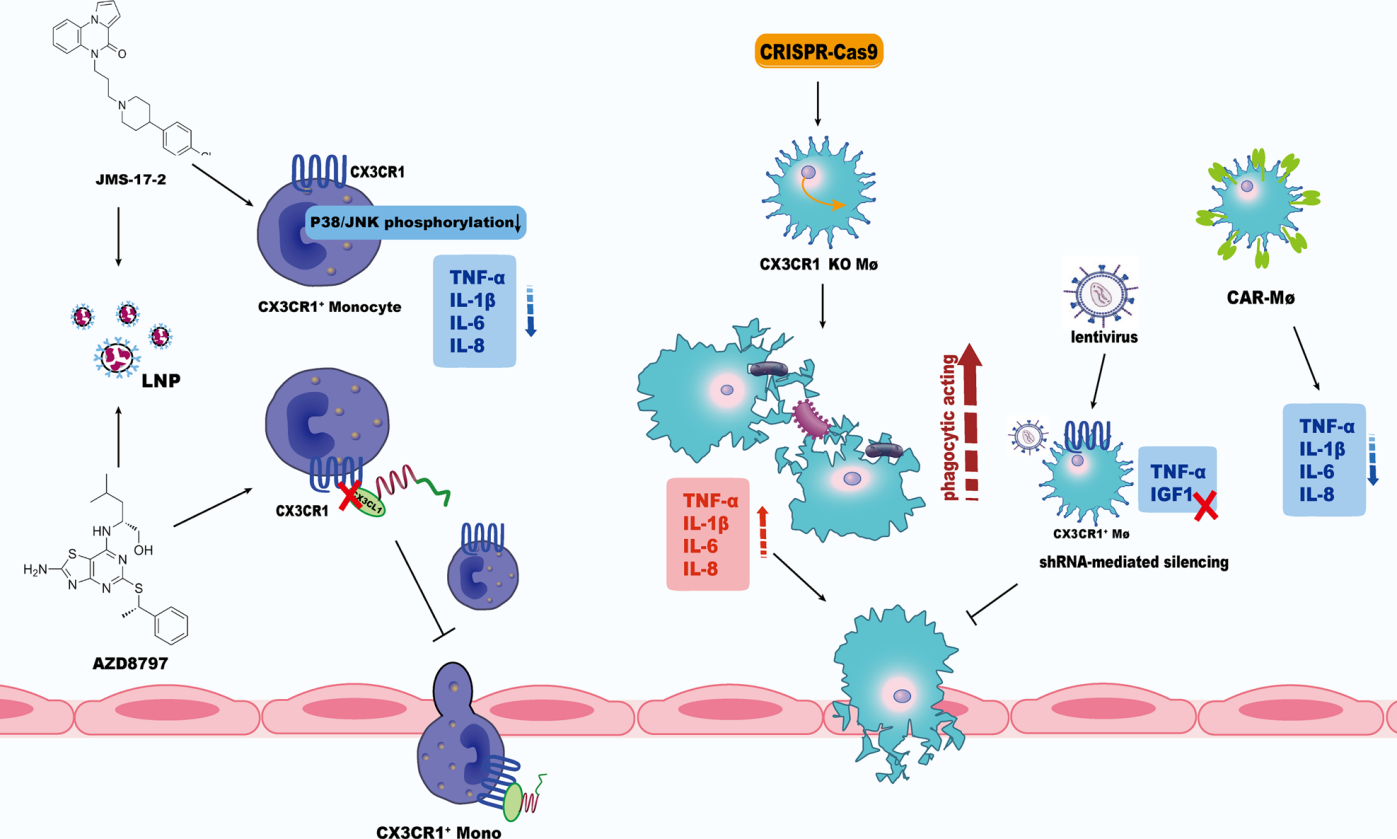
Strategies targeting CX3CR1 in monocytes and macrophages. Multiple approaches can modulate CX3CR1 signaling in the monocyte-macrophage system. Pharmacological inhibition using JMS-17-2 or AZD8797 suppresses inflammatory cytokine release or monocyte recruitment. LNP-mediated delivery may improve targeting efficiency. Gene-editing interventions, including CRISPR-Cas9 knockout or lentivirus-mediated silencing of TNF-α and IGF1 in CX3CR1⁺ macrophages, regulate phagocytic and inflammatory activity. Engineered CAR macrophages (CAR-Ms) can exert anti-inflammatory and tissue-repair functions, underscoring the therapeutic potential of CX3CR1⁺ CAR-Ms in sepsis.

Regarding cellular therapies, CAR macrophages (CAR-Ms) are recently engineered to respond to inflammatory cytokines for the treatment of inflammatory diseases across various organs. Upon activation by cytokines in injured tissue, CAR-Ms elicit anti-inflammatory and tissue-repair responses, effectively resolving inflammation and reducing tissue damage. These findings highlight the therapeutic potential of CX3CR1⁺ CAR-Ms in sepsis [121] (Fig. 4).

### Potential T cell-based immunomodulatory strategies

**Fig. 5:**
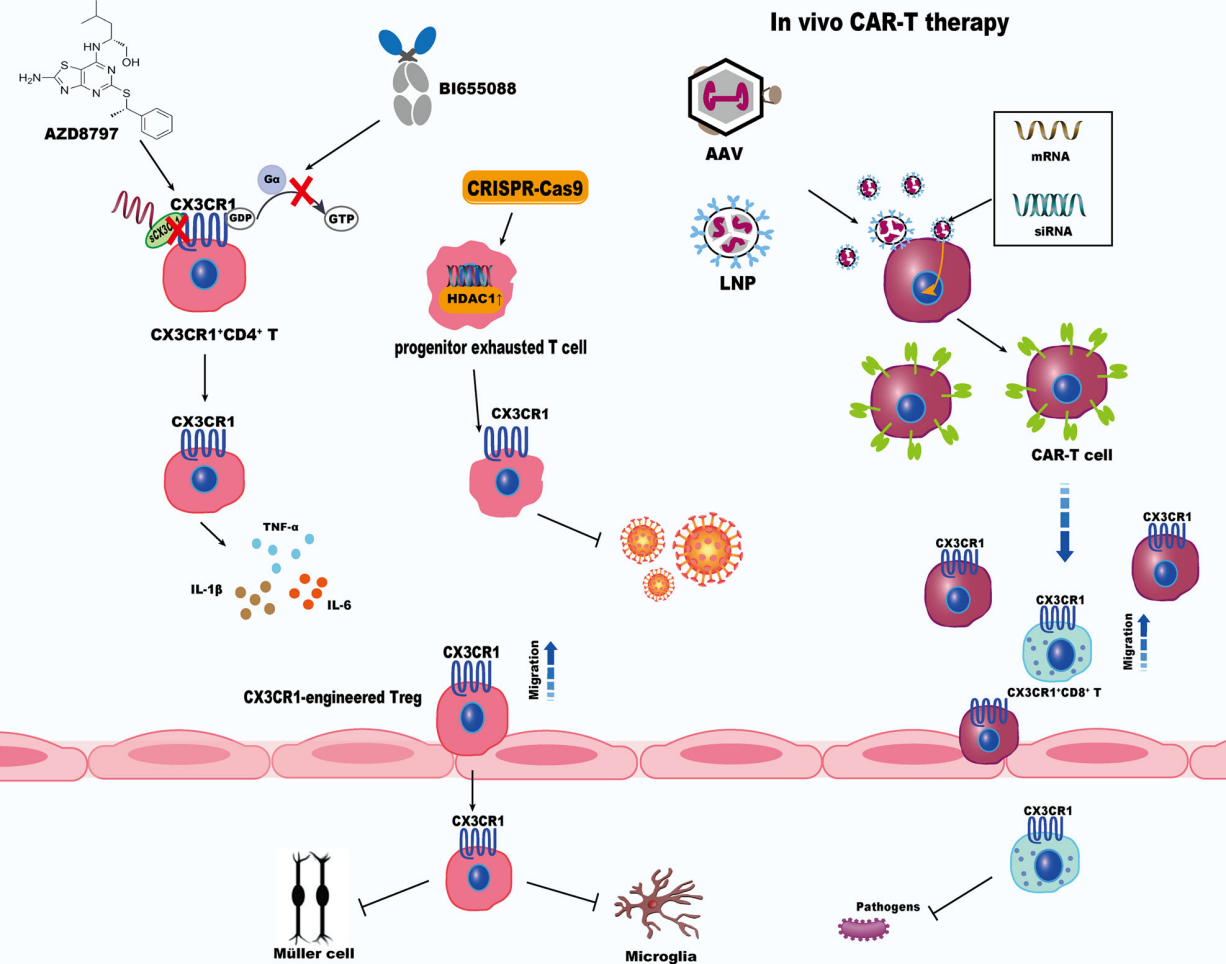
Strategies targeting CX3CR1 in T cells. CX3CR1 signaling in T cells can be modulated through multiple approaches to restore immune balance during sepsis. Pharmacological inhibitor or antibody (e.g., AZD8797, BI655088) reduces CX3CR1 activation on CD4⁺ T cells and attenuates cytokine release. Gene editing of HDAC1 via CRISPR-Cas9 enhances differentiation of progenitor exhausted T cells toward CX3CR1⁺ subsets, improving effector function. CX3CR1-engineered Tregs migrate to inflamed tissues and suppress microglial activation. Furthermore, in vivo CAR-T cell engineering using LNPs or AAV enables targeted delivery of mRNA/siRNA, providing a promising avenue for CX3CR1⁺ T cell–based immunotherapy in sepsis.

The pivotal role of CX3CR1 in T cell subsets offers new insights into immune modulation during sepsis. CX3CR1-engineered Tregs have been shown to target activated microglia and alleviate neuroinflammation, an approach potentially applicable to the systemic inflammatory regulation in sepsis [111]. Rica et al. showed that histone deacetylase 1 (HDAC1) facilitates an open chromatin state at effector-associated gene loci in progenitor exhausted T cells, driving their differentiation toward the CX3CR1⁺ subset, which plays a key role in viral control during infection. This implies that HDAC1 upregulation through gene editing could reverse T cell exhaustion in sepsis [108]. Moreover, recent advances have combined CAR-T technology with gene editing to deliver mRNA or siRNA into T cells using targeted LNPs or adeno-associated virus, enabling in vivo T cell engineering—a promising strategy for treating sepsis [122, 123] (Fig. 5).

## Translational challenges and future perspectives

Currently, CX3CR1-targeted therapies face multiple translational challenges. Drug development has been slow, with only a few candidates reaching clinical trials. To date, a high-affinity small-molecule inhibitor, AZD8797 [19, 20], and a CX3CR1-specific nanobody, BI 655088, have entered clinical trials [22] for the treatment of cancer-associated pain and kidney disease, respectively (Table 1).

**Table 1:** Pharmacotherapy directly or indirectly targeting the CX3CL1–CX3CR1 axis.

Target	Name	Structure	Effect	Mechanism	Cell	Species	Disease	Clincal trial	Reference
CX3CR1	E6130		induced down-regulation of CX3CR1 on the cell surface of NK cells	inhibit the migration of CX3CR1^+^ immune cells and decrease the number of these cells in the gut mucosal membrane	NK cells	mouse	inflammatory bowel disease	NA	Wakita et al.
CX3CR1	JMS172		selective CX3CR1 antagonist	reduce pro-inflammatory cytokine production in monocytes by inhibiting p38/JNK phosphorylation	monocytes	mouse	depression; preeclampsia	NA	Liu et al; Qin et al; Goode-Romero et al.
CX3CL1	AZD8797/Rugocrixan/KAND567		orally bioavailable, single high-affinity, allosteric modulator of CX3CL1 that binds non-competitively to CX3CR1 and interferes with downstream G-protein signaling	disrupt the CX3CL1–CX3CR1 axis and suppress monocyte recruitment to sites of inflammation; limit leukocyte infiltration into the central nervous system; reduce microglial activation and migration, and suppress Müller cell gliosis	monocytes; leukocytes; Müller cell	mouse, human	retinal degeneration; neuroinflammation; Kainic Acid-Induced Status Epilepticus; cancer; hepatic ischemia–reperfusion injury	phase II	Cederblad et al; Huang et al; Shih et al.
CX3CR1	BI655088	NA	block the CX3CR1 receptor in vivo	inhibit plaque progression in the standard mouse model of atherosclerosis	NA	mouse, monkey, human	atherosclerosis; kidney disease	phase I	Low et al;
CX3CR1	CX3CR1-specific monoclonal antibody clones 455.1C11, and 455.8H12	NA	inhibit the CX3CL1-CX3CR1 interaction	reduce tumor cell migration and the release of immunosuppressive mediators	myeloid cell	mouse	CT26 tumor cell line; colon carcinoma	NA	Chaudhri et al.
CX3CR1	MSCs	NA	recruit CX3CR1^high^ macrophages and promote their M1 polarization to inhibit tumor growth via highly secretion of CX3CL1	enhance the homing capacity of macrophages to inflamed tissues	MSCs; macrophages	mouse	colorectal cancer	NA	Liu et al.
CX3CR1	CRISPR-Cas9-mediated knockout of CX3CR1	NA	deletes CX3CR1 in human iPS cells	increased inflammatory responses and phagocytic activity in mutant as compared to wild-type microglia-like cells	human induced pluripotent stem (iPS) cell-derived microglia-like cells	human	neurodegenerative diseases and neuroinflammatory disorders	NA	Murai et al.
CX3CR1	CX3CR1-transduced Tregs	NA	modulate microglial activity and inhibit LPS-induced neuroinflammation	reduce proinflammatory marker expression in both the cortices and hippocampi	Tregs	mouse	Alzheimer’s disease	NA	Yang et al.
CX3CR1	the key epigenetic regulator histone deacetylase 1 (HDAC1)	NA	increase the CX3CR1⁺ Tex subset	facilitate an open chromatin configuration at effector-associated gene loci in progenitor exhausted T cells, thereby promote the generation and maintenance of effector-like CX3CR1^+^ Tex cells in a CD8^+^ T cell-intrinsic manner	T cells	mouse	chronic viral infection	NA	Rica et al.
CX3CR1	sini decoction (SND)	NA	modulate CX3CR1-related signaling pathways	alleviate inflammation, improve microcirculation, and modulate immune status in sepsis patients	NA	mouse, human	sepsis	Applicable	Gu et al.

AZD8797, an orally bioavailable allosteric modulator of CX3CL1, binds non-competitively to CX3CR1 and disrupts downstream G-protein signaling. It reduces neuroinflammation by suppressing the release of proinflammatory cytokines such as IL-1β, IL-6, and TNF-α, and limits leukocyte infiltration into the CNS [124, 125]. In retinal degeneration models, AZD8797 preserved retinal structure, inhibited microglial activation and migration, and suppressed Müller cell gliosis, resulting in improved retinal function, as confirmed by behavioral tests and electroretinography [21]. Its efficacy in models of neuroinflammation [42], hepatic ischemia–reperfusion injury [11], and cancer [126] supports its translational potential for sepsis therapy.

CX3CR1-targeting antibodies have shown early clinical promise in tumor immunology. Chaudhri et al. suggested that a monoclonal antibody blocking CX3CL1-CX3CR1 interaction inhibited tumor cell migration and reduced the release of immunosuppressive mediators. When combined with anti-PD-1 immunotherapy, this antibody improved survival by limiting tumor-promoting myeloid cell infiltration in a murine colon carcinoma model [28]. Additionally, the CX3CR1-specific nanobody BI 655088 has entered Phase I clinical trials for the treatment of kidney diseases [22]. These findings highlight the potential of CX3CR1 as a immunotherapeutic target in sepsis.

In gene therapy, although CRISPR-Cas9 technology shows substantial promise, challenges remain regarding low vector delivery efficiency, high immunogenicity, and uncertain long-term safety profiles [127–129]. In addition, recent advances in nanocarrier technology have shown great translational potential and may help overcome delivery challenges in gene therapy for sepsis [130, 131].

In cell-based therapies, CX3CR1 gene engineering has been shown to enhance the homing capacity of mesenchymal stem cells (MSCs) to inflamed tissues, suggesting that similar strategies could be applied to modify T cells or macrophages for CAR-T or CAR-M therapies. However, large-scale production and personalized application remain major challenges in the field of cell therapy [132].

However, most evidence supporting CX3CR1 modulation originates from non-sepsis disease models, while sepsis-specific interventional data remain limited. Validation in sepsis-specific models and consideration of translational relevance are needed. In addition, any therapeutic strategy targeting the CX3CR1-mediated signaling pathway should carefully consider the disease stage, as uniform inhibition or activation during either the hyperinflammatory or immunosuppressive phase may carry potential risks.

## Conclusions

In summary, CX3CR1 exerts diverse functions across multiple immune cell types through context- and cell-specific mechanisms. A deeper understanding of its immunoinflammatory pathways, combined with targeted and personalized modulation strategies, holds great promise for advancing precision therapies in sepsis.

## References

[1] Xie J, Wang H, Kang Y, Zhou L, Liu Z, Qin B, et al. The epidemiology of sepsis in chinese ICUs: a national cross-sectional survey. Crit Care Med. 2020;48:e209–e18. 31804299 10.1097/CCM.0000000000004155

[2] van der Poll T, Shankar-Hari M, Wiersinga WJ. The immunology of sepsis. Immunity. 2021;54:2450–64. 34758337 10.1016/j.immuni.2021.10.012

[3] Singer M, Deutschman CS, Seymour CW, Shankar-Hari M, Annane D, Bauer M, et al. The third international consensus definitions for sepsis and septic shock (Sepsis-3). JAMA. 2016;315:801–10. 10.1001/jama.2016.0287 PMC4968574 26903338

[4] Willmann K, Moita LF. Physiologic disruption and metabolic reprogramming in infection and sepsis. Cell Metab. 2024;36:927–46. 38513649 10.1016/j.cmet.2024.02.013

[5] Vincent JL, van der Poll T, Marshall JC. The end of “One Size Fits All” sepsis therapies: Toward an individualized approach. Biomedicines. 2022;10:2260. 10.3390/biomedicines10092260 PMC9496597 36140361

[6] Rao M, McGonagill PW, Brackenridge S, Remy KE, Caldwell CC, Hotchkiss RS, et al. Functional immunophenotyping for precision therapies in sepsis. Shock. 2025;63:189–201. 39617419 10.1097/SHK.0000000000002511 PMC12447363

[7] Darden DB, Kelly LS, Fenner BP, Moldawer LL, Mohr AM, Efron PA. Dysregulated immunity and immunotherapy after sepsis. J Clin Med. 2021;10:1742. 10.3390/jcm10081742 PMC8073536 33920518

[8] Prucha M, Zazula R, Russwurm S. Immunotherapy of sepsis: Blind alley or call for personalized assessment?. Arch Immunol Ther Exp (Warsz). 2017;65:37–49. 27554587 10.1007/s00005-016-0415-9

[9] Steinhagen F, Schmidt SV, Schewe JC, Peukert K, Klinman DM, Bode C. Immunotherapy in sepsis - brake or accelerate?. Pharm Ther. 2020;208:107476. 10.1016/j.pharmthera.2020.107476 31931100

[10] Ma J, Wu Y, Wu S, Fang Z, Chen L, Jiang J, et al. CX3CR1^+^CD8^+^ T cells: Key players in antitumor immunity. Cancer Sci. 2024;115:3838–45. 39377122 10.1111/cas.16359 PMC11611776

[11] Zhang H, You G, Yang Q, Jin G, Lv G, Fan L, et al. CX3CR1 deficiency promotes resolution of hepatic ischemia-reperfusion injury by regulating homeostatic function of liver infiltrating macrophages. Biochim Biophys Acta (BBA) - Mol Basis Dis. 2024;1870:167130. 10.1016/j.bbadis.2024.167130 38537684

[12] Qian X, Zheng Y, Xu L, Liu Z, Chen M, Tong F, et al. Deciphering the role of CX3CL1-CX3CR1 in aortic aneurysm pathogenesis: insights from Mendelian randomization and transcriptomic analyses. Front Immunol. 2024;15:1383607. 10.3389/fimmu.2024.1383607 PMC11074460 38715600

[13] Chen X, Yang Y, Sun S, Liu Q, Yang Y, Jiang L. CX3C chemokine: Hallmarks of fibrosis and ageing. Pharmacol Res. 2024;208:107348. 10.1016/j.phrs.2024.107348 39134186

[14] Moon H-G, Kim S-J, Kim K-H, Kim Y-M, Rehman J, Lee H, et al. CX3CR1^+^ macrophage facilitates the resolution of allergic lung inflammation via interacting CCL26. Am J Respir Crit Care Med. 2023;207:1451–63. 36790376 10.1164/rccm.202209-1670OC PMC10263139

[15] El-Shazly AE, Doloriert HC, Bisig B, Lefebvre PP, Delvenne P, Jacobs N. Novel cooperation between CX3CL1 and CCL26 inducing NK cell chemotaxis via CX3CR1: a possible mechanism for NK cell infiltration of the allergic nasal tissue. Clin Exp Allergy. 2013;43:322–31. 23414540 10.1111/cea.12022

[16] Nakayama T, Watanabe Y, Oiso N, Higuchi T, Shigeta A, Mizuguchi N, et al. Eotaxin-3/CC chemokine ligand 26 is a functional ligand for CX3CR1. J Immunol. 2010;185:6472–9. 20974991 10.4049/jimmunol.0904126

[17] Li BH, Garstka MA, Li ZF. Chemokines and their receptors promoting the recruitment of myeloid-derived suppressor cells into the tumor. Mol Immunol. 2020;117:201–15. 31835202 10.1016/j.molimm.2019.11.014

[18] Lu MZW, Han S, Lin X, Xu T, Tan Q, Wang M, Yi C, Chu X, Yang W, Zhu Y, Wu B, Zhao Q. Activation of the human chemokine receptor CX3CR1 regulated by cholesterol. Sci Adv. 2022;8:eabn8048. 35767622 10.1126/sciadv.abn8048 PMC9242592

[19] Chen L, Zhang L, Jin G, Liu Y, Guo N, Sun H, et al. Synergy of 5-aminolevulinate supplement and CX3CR1 suppression promotes liver regeneration via elevated IGF-1 signaling. Cell Rep. 2023;42:112984. 37578861 10.1016/j.celrep.2023.112984

[20] Cederblad L, Rosengren B, Ryberg E, Hermansson NO. AZD8797 is an allosteric non-competitive modulator of the human CX3CR1 receptor. Biochem J. 2016;473:641–9. 26656484 10.1042/BJ20150520 PMC4764977

[21] Huang JM, Zhao N, Hao XN, Li SY, Wei D, Pu N, et al. CX3CL1/CX3CR1 signaling mediated neuroglia activation is implicated in the retinal degeneration: A potential therapeutic target to prevent photoreceptor death. Investig Ophthalmol Vis Sci. 2024;65:29. 10.1167/iovs.65.1.29 PMC10795588 38231527

[22] Low S, Wu H, Jerath K, Tibolla A, Fogal B, Conrad R, et al. VHH antibody targeting the chemokine receptor CX3CR1 inhibits progression of atherosclerosis. MAbs. 2020;12:1709322. 31924119 10.1080/19420862.2019.1709322 PMC6973309

[23] Liu X, Yu Z, Li Y, Huang J. CX3CL1 and its receptor CX3CR1 interact with RhoA signaling to induce paclitaxel resistance in gastric cancer. Heliyon. 2024;10:e29100. 38601629 10.1016/j.heliyon.2024.e29100 PMC11004636

[24] Goode-Romero G, Dominguez L. Computational study of the conformational ensemble of CX3C chemokine receptor 1 (CX3CR1) and its interactions with antagonist and agonist ligands. J Mol Graph Model. 2022;117:108278. 35988439 10.1016/j.jmgm.2022.108278

[25] Lee M, Lee Y, Song J, Lee J, Chang S-Y. Tissue-specific role of CX3CR1 expressing immune cells and their relationships with human disease. Immune Netw. 2018;18:e5. 10.4110/in.2018.18.e5 PMC5833124 29503738

[26] Shah R, Matthews GJ, Shah RY, McLaughlin C, Chen J, Wolman M, et al. Serum fractalkine (CX3CL1) and cardiovascular outcomes and diabetes: findings from the chronic renal insufficiency cohort (CRIC) study. Am J Kidney Dis. 2015;66:266–73. 25795074 10.1053/j.ajkd.2015.01.021 PMC4516570

[27] Iemmolo M, Ghersi G, Bivona G. The cytokine CX3CL1 and ADAMs/MMPs in concerted cross-talk influencing neurodegenerative diseases. Int J Mol Sci. 2023;24:8026. 10.3390/ijms24098026 PMC10179166 37175729

[28] Chaudhri A, Bu X, Wang Y, Gomez M, Torchia JA, Hua P, et al. The CX3CL1-CX3CR1 chemokine axis can contribute to tumor immune evasion and blockade with a novel CX3CR1 monoclonal antibody enhances response to anti-PD-1 immunotherapy. Front Immunol. 2023;14:1237715. 37771579 10.3389/fimmu.2023.1237715 PMC10524267

[29] Imai T, Yasuda N. Therapeutic intervention of inflammatory/immune diseases by inhibition of the fractalkine (CX3CL1)-CX3CR1 pathway. Inflamm Regen. 2016;36:9. 29259682 10.1186/s41232-016-0017-2 PMC5725656

[30] Subbarayan MS, Joly-Amado A, Bickford PC, Nash KR. CX3CL1/CX3CR1 signaling targets for the treatment of neurodegenerative diseases. Pharm Ther. 2022;231:107989. 10.1016/j.pharmthera.2021.107989 34492237

[31] Chen X, Wei Q, Hu Y, Wang C. Role of Fractalkine in promoting inflammation in sepsis-induced multiple organ dysfunction. Infect Genet Evol. 2020;85:104569. 32979549 10.1016/j.meegid.2020.104569

[32] Fraticelli PSM, Bianchi G, D’Ambrosio D, Albanesi C, Stoppacciaro A, Chieppa M, Allavena P, Ruco L, Girolomoni G, Sinigaglia F, Vecchi A, Mantovani A. Fractalkine (CX3CL1) as an amplification circuit of polarized Th1 responses. J Clin Investig. 2001;107:1173–81. 11342581 10.1172/JCI11517 PMC209276

[33] Li C, Zhang Z, Cai Q, Zhao Q, Wu H, Li J, et al. Peripheral CX3CR1^+^ T cells combined with PD-1 blockade therapy potentiates the anti-tumor efficacy for lung cancer. Oncoimmunology. 2024;13:2355684. 38798746 10.1080/2162402X.2024.2355684 PMC11123541

[34] White GE, Greaves DR. Fractalkine: a survivor’s guide: chemokines as antiapoptotic mediators. Arterioscler Thromb Vasc Biol. 2012;32:589–94. 22247260 10.1161/ATVBAHA.111.237412

[35] Qin M, Chen C, Wang N, Yu D, Yu S, Wang X, et al. Total saponins of panax ginseng via the CX3CL1/CX3CR1 axis attenuates neuroinflammation and exerted antidepressant-like effects in chronic unpredictable mild stress in rats. Phytother Res. 2023;37:1823–38. 36581492 10.1002/ptr.7696

[36] Iwahashi Y, Ishida Y, Mukaida N, Kondo T. Pathophysiological roles of the CX3CL1-CX3CR1 axis in renal disease, cardiovascular disease, and cancer. Int J Mol Sci. 2025;26:5352. 10.3390/ijms26115352 PMC12155443 40508161

[37] Gao XW, Hu HL, Xie MH, Tang CX, Ou J, Lu ZH. CX3CL1/CX3CR1 axis alleviates inflammation and apoptosis in human nucleus pulpous cells via M2 macrophage polarization. Exp Ther Med. 2023;26:359. 37324510 10.3892/etm.2023.12058 PMC10265713

[38] Li Q, Yuan Z, Bahabayi A, Zhang Z, Zeng X, Kang R, et al. Upregulation of CX3CR1 expression in circulating T cells of systemic lupus erythematosus patients as a reflection of autoimmune status through characterization of cytotoxic capacity. Int Immunopharmacol. 2024;126:111231. 38016349 10.1016/j.intimp.2023.111231

[39] Babcock IW, Sibley LA, Labuzan SA, Cowan MN, Sethi I, Alemu S, et al. Caspase-1 in CX3CR1-expressing cells drives an IL-18-dependent T cell response that promotes parasite control during acute Toxoplasma gondii infection. PLoS Pathog. 2024;20:e1012006. 39446964 10.1371/journal.ppat.1012006 PMC11537422

[40] Pokharel J, Shryki I, Zwijnenburg AJ, Sandu I, Krumm L, Bekiari C, et al. The cellular microenvironment regulates CX3CR1 expression on CD8^+^ T cells and the maintenance of CX3CR1^+^ CD8^+^ T cells. Eur J Immunol. 2024;54:e2350658. 37816219 10.1002/eji.202350658

[41] White GE, Tan TC, John AE, Whatling C, McPheat WL, Greaves DR. Fractalkine has anti-apoptotic and proliferative effects on human vascular smooth muscle cells via epidermal growth factor receptor signalling. Cardiovasc Res. 2010;85:825–35. 19840952 10.1093/cvr/cvp341 PMC2819832

[42] Chen X, He X, Xu F, Xu N, Sharifi NH, Zhang P, et al. Fractalkine enhances hematoma resolution and improves neurological function via CX3CR1/AMPK/PPARgamma pathway after GMH. Stroke. 2023;54:2420–33. 37465997 10.1161/STROKEAHA.123.043005 PMC10453335

[43] Akiyama MYK, Kaneko Y. Significant association of CX3CR1^+^ CD8 T cells with aging and distinct clinical features in Sjögren’s syndrome and IgG4-related disease. Clin Exp Rheumatol. 2023;41:2409–17. 37812481 10.55563/clinexprheumatol/kfsd65

[44] Landsman L, Bar-On L, Zernecke A, Kim KW, Krauthgamer R, Shagdarsuren E, et al. CX3CR1 is required for monocyte homeostasis and atherogenesis by promoting cell survival. Blood. 2009;113:963–72. 18971423 10.1182/blood-2008-07-170787

[45] Lu Z, Zhang A, Dai Y. CX3CL1 deficiency ameliorates inflammation, apoptosis and accelerates osteogenic differentiation, mineralization in LPS-treated MC3T3-E1 cells via its receptor CX3CR1. Ann Anat. 2023;246:152036. 36436718 10.1016/j.aanat.2022.152036

[46] Li T, Li X, Kang P, Zhao J. Exploring CX3CR1 as a prognostic biomarker and immunotherapeutic target in sarcoma. Transl Oncol. 2025;53:102283. 39837057 10.1016/j.tranon.2025.102283 PMC11787715

[47] Gu Y, Li Z, Li H, Yi X, Liu X, Zhang Y, et al. Exploring the efficacious constituents and underlying mechanisms of sini decoction for sepsis treatment through network pharmacology and multi-omics. Phytomedicine. 2024;123. 10.1016/j.phymed.2023.155212 38029626

[48] Ishida YHT, Goto T, Kimura A, Akimoto S, Mukaida N, Kondo T. Essential involvement of CX3CR1-mediated signals in the bactericidal host defense during septic peritonitis. J Immunol. 2008;181:4208–18. 18768878 10.4049/jimmunol.181.6.4208

[49] Zhuang Q, Cheng K, Ming Y. CX3CL1/CX3CR1 axis, as the therapeutic potential in renal diseases: friend or foe?. Curr Gene Ther. 2017;17:442–52. 29446734 10.2174/1566523218666180214092536 PMC5902862

[50] Huang LY, Chen P, Xu LX, Zhou YF, Zhang YP, Yuan YZ. Fractalkine upregulates inflammation through CX3CR1 and the Jak-Stat pathway in severe acute pancreatitis rat model. Inflammation. 2012;35:1023–30. 22213034 10.1007/s10753-011-9406-5

[51] Hassan NF, El-Ansary MR, Selim H, Ousman MS, Khattab MS, El-Ansary MRM, et al. Alirocumab boosts antioxidant status and halts inflammation in rat model of sepsis-induced nephrotoxicity via modulation of Nrf2/HO-1, PCSK9/HMGB1/NF-ᴋB/NLRP3 and Fractalkine/CX3CR1 hubs. Biomed Pharmacother. 2024;177:116929. 38889644 10.1016/j.biopha.2024.116929

[52] Hamdan D, Robinson LA. Role of the CX(3)CL1-CX(3)CR1 axis in renal disease. Am J Physiol Ren Physiol. 2021;321:F121–F34. 10.1152/ajprenal.00059.2021 34121453

[53] Pachot A, Cazalis MA, Venet F, Turrel F, Faudot C, Voirin N, et al. Decreased expression of the fractalkine receptor CX3CR1 on circulating monocytes as new feature of sepsis-induced immunosuppression. J Immunol. 2008;180:6421–9. 18424766 10.4049/jimmunol.180.9.6421

[54] Ge XYFS, Zhou M, Luo J, Wei J, Wen XP, Yan XD, Zou Z. TLR4-dependent internalization of CX3CR1 aggravates sepsis-induced immunoparalysis. Am J Transl Res. 2016;8:5696–705. 28078040 PMC5209520

[55] Raspe C, Hocherl K, Rath S, Sauvant C, Bucher M. NF-kappaB-mediated inverse regulation of fractalkine and CX3CR1 during CLP-induced sepsis. Cytokine. 2013;61:97–103. 23026294 10.1016/j.cyto.2012.08.034

[56] Ramos-Hernandez I, Fuster-Garcia C, Aguilar-Gonzalez A, Lozano-Vinagre ML, Guenechea-Amurrio G, Sanchez-Luque FJ, et al. Donor insertion into CX3CR1 allows epigenetic modulation of a constitutive promoter on hematopoietic stem cells and its activation upon myeloid differentiation. Nucleic Acids Res. 2025;53:gkaf344. 10.1093/nar/gkaf344 PMC12038399 40298109

[57] He Y, Dong S, Ouyang L, Kang J, Chen L, He Q. Integration of single-cell and RNA-seq analysis reveals sepsis heterogeneity and prognostic significance of FCGR3A^+^ macrophage subtypes. Biochem Biophys Rep. 2025;44:102352. 41332909 10.1016/j.bbrep.2025.102352 PMC12666706

[58] Cazalis MA, Kreitmann L, Monneret G, Pachot A, Brengel-Pesce K, Llitjos JF. Whole blood ratio of CDK1/CX3CR1 mRNA expression combined to lactate refines the prediction of ICU mortality in septic patients in the Sepsis-3 era: a proof-of-concept study. Front Med. 2024;11:1445451. 10.3389/fmed.2024.1445451 PMC11739359 39830374

[59] Friggeri A, Cazalis MA, Pachot A, Cour M, Argaud L, Allaouchiche B, et al. Decreased CX3CR1 messenger RNA expression is an independent molecular biomarker of early and late mortality in critically ill patients. Crit Care. 2016;20:204. 27364780 10.1186/s13054-016-1362-x PMC4929760

[60] Jiang J, Chen Y, Su Y, Zhang L, Qian H, Song X, et al. Identification and experimental validation of diagnostic and prognostic genes CX3CR1, PID1 and PTGDS in sepsis and ARDS using bulk and single-cell transcriptomic analysis and machine learning. Front Immunol. 2024;15:1480542. 39763654 10.3389/fimmu.2024.1480542 PMC11700820

[61] Yang L, Gao Q, Li Q, Guo S. PD-L1 blockade improves survival in sepsis by reversing monocyte dysfunction and immune disorder. Inflammation. 2024;47:114–28. 37776443 10.1007/s10753-023-01897-0 PMC10799109

[62] Chen L, Zhang X, Shi P. Recent advances in biomarkers for detection and diagnosis of sepsis and organ dysfunction: a comprehensive review. Eur J Med Res. 2025;30:1081. 41204362 10.1186/s40001-025-03324-6 PMC12595651

[63] Ancuta P, Rao R, Moses A, Mehle A, Shaw SK, Luscinskas FW, et al. Fractalkine preferentially mediates arrest and migration of CD16^+^ monocytes. J Exp Med. 2003;197:1701–7. 12810688 10.1084/jem.20022156 PMC2193954

[64] Wu H, Han Y, Song Z, Li W, Ke P, Wu X. Genetic evidence reveals a causal association between plasma phosphatidylcholine levels and sepsis associated mortality, with immune cells mediating this association. Shock. 2026;65:597-604. 10.1097/SHK.0000000000002617 40333460

[65] Pi D, Wong JJM, Nay Yaung K, Khoo NKH, Poh SL, Wasser M, et al. Clinical and mechanistic relevance of high-dimensionality analysis of the paediatric sepsis immunome. Front Immunol. 2025;16:1569096. 10.3389/fimmu.2025.1569096 PMC12106532 40433376

[66] Siddiqui I, Erreni M, van Brakel M, Debets R, Allavena P. Enhanced recruitment of genetically modified CX3CR1-positive human T cells into Fractalkine/CX3CL1 expressing tumors: importance of the chemokine gradient. J. ImmunoTher. Cancer. 2016;4:21. 10.1186/s40425-016-0125-1 PMC4836203 27096098

[67] Łyszkiewicz M, Witzlau K, Pommerencke J, Krueger A. Chemokine receptor CX3CR1 promotes dendritic cell development under steady-state conditions. Eur J Immunol. 2011;41:1256–65. 21425158 10.1002/eji.201040977

[68] Ni Y, Zhuge F, Ni L, Nagata N, Yamashita T, Mukaida N, et al. CX3CL1/CX3CR1 interaction protects against lipotoxicity-induced nonalcoholic steatohepatitis by regulating macrophage migration and M1/M2 status. Metabolism. 2022;136:155272. 35914622 10.1016/j.metabol.2022.155272

[69] Montepeloso A, Mattioli D, Pellin D, Peviani M, Genovese P, Biffi A. Haploinsufficiency at the CX3CR1 locus of hematopoietic stem cells favors the appearance of microglia-like cells in the central nervous system of transplant recipients. Nat Commun. 2024;15:10192. 39587072 10.1038/s41467-024-54515-4 PMC11589136

[70] Ensan S, Li A, Besla R, Degousee N, Cosme J, Roufaiel M, et al. Self-renewing resident arterial macrophages arise from embryonic CX3CR1(+) precursors and circulating monocytes immediately after birth. Nat Immunol. 2016;17:159–68. 26642357 10.1038/ni.3343

[71] Collar AL, Swamydas M, O’Hayre M, Sajib MS, Hoffman KW, Singh SP, et al. The homozygous CX3CR1-M280 mutation impairs human monocyte survival. JCI Insight. 2018;3:e95417. 10.1172/jci.insight.95417 PMC5821174 29415879

[72] Zhang YZL, Wang X, Ma W, Lazere A, Qian HH, Zhang J, Abu-Asab M, Fariss RN, Roger JE, Wong WT. Repopulating retinal microglia restore endogenous organization and function under CX3CL1-CX3CR1 regulation. Sci Adv. 2018;4:eaap8492. 29750189 10.1126/sciadv.aap8492 PMC5943055

[73] Yang Z, Liu M, Chang Z, Du C, Yang Y, Zhang C, et al. Myeloid-derived growth factor promotes M2 macrophage polarization and attenuates Sjogren’s syndrome via suppression of the CX3CL1/CX3CR1 axis. Front Immunol. 2024;15:1465938. 39497829 10.3389/fimmu.2024.1465938 PMC11532040

[74] Chousterman BG, Boissonnas A, Poupel L, Baudesson de Chanville C, Adam J, Tabibzadeh N, et al. Ly6Chigh monocytes protect against kidney damage during sepsis via a CX3CR1-dependent adhesion mechanism. J Am Soc Nephrol. 2016;27:792–803. 26160897 10.1681/ASN.2015010009 PMC4769199

[75] Zhan L, Qiu M, Zheng J, Lai M, Lin K, Dai J, et al. Fractalkine/CX3CR1 axis is critical for neuroprotection induced by hypoxic postconditioning against cerebral ischemic injury. Cell Commun Signal. 2024;22:457. 39327578 10.1186/s12964-024-01830-4 PMC11426015

[76] Han C, Zhai Y, Wang Y, Peng X, Zhang X, Dai B, et al. Intravital imaging of splenic classical monocytes modifying the hepatic CX3CR1^+^ cells motility to exacerbate liver fibrosis via spleen-liver axis. Theranostics. 2024;14:2210–31. 38505603 10.7150/thno.87791 PMC10945343

[77] An JL, Ishida Y, Kimura A, Tsokos M, Kondo T. Immunohistochemical detection of CCR2 and CX3CR1 in sepsis-induced lung injury. Forensic Sci Int. 2009;192:e21–5. 19733456 10.1016/j.forsciint.2009.08.007

[78] Alarcon-Sanchez MA, Becerra-Ruiz JS, Guerrero-Velazquez C, Mosaddad SA, Heboyan A. The role of the CX3CL1/CX3CR1 axis as potential inflammatory biomarkers in subjects with periodontitis and rheumatoid arthritis: a systematic review. Immun Inflamm Dis. 2024;12:e1181. 38415821 10.1002/iid3.1181 PMC10845211

[79] Pezeshkian F, Shahriarirad R, Mahram H. An overview of the role of chemokine CX3CL1 (Fractalkine) and CX3C chemokine receptor 1 in systemic sclerosis. Immun Inflamm Dis. 2024;12:e70034. 39392260 10.1002/iid3.70034 PMC11467895

[80] Zhang J, Fang Q, Huang Y, Qu Y, Liu Q, Li R, et al. CX3CR1(+) monocytes/macrophages promote regional immune injury in mesangial proliferative glomerulonephritis through crosstalk with activated mesangial cells. Research. 2025;8:0716. 40458609 10.34133/research.0716 PMC12128197

[81] Zhou Z, Zhang H, Tao Y, Jie H, Zhao J, Zang J, et al. CX3CR1(hi) macrophages sustain metabolic adaptation by relieving adipose-derived stem cell senescence in visceral adipose tissue. Cell Rep. 2023;42:112424. 37086405 10.1016/j.celrep.2023.112424

[82] Xiang XWK, Zhang H, Mou H, Shi Z, Tao Y, Song H, Lian Z, Wang S, Lu D, Wei X, Xie H, Zheng S, Wang J, Xu X. Blocking CX3CR1^+^ tumor-associated macrophages enhances the efficacy of anti-PD1 therapy in hepatocellular carcinoma. Cancer Immunol Res. 2024;12:1603–20. 39115356 10.1158/2326-6066.CIR-23-0627

[83] Lauro C, Chece G, Monaco L, Antonangeli F, Peruzzi G, Rinaldo S, et al. Fractalkine modulates microglia metabolism in brain ischemia. Front Cell Neurosci. 2019;13:414. 31607865 10.3389/fncel.2019.00414 PMC6755341

[84] Pandur E, Tamasi K, Pap R, Janosa G, Sipos K. Modulatory effects of fractalkine on inflammatory response and iron metabolism of lipopolysaccharide and lipoteichoic acid-activated THP-1 macrophages. Int J Mol Sci. 2022;23:2629. 10.3390/ijms23052629 PMC8910483 35269771

[85] Puntambekar SS, Moutinho M, Lin PB, Jadhav V, Tumbleson-Brink D, Balaji A, et al. CX3CR1 deficiency aggravates amyloid driven neuronal pathology and cognitive decline in Alzheimer’s disease. Mol Neurodegener. 2022;17:47. 35764973 10.1186/s13024-022-00545-9 PMC9241248

[86] Lecordier S, Menet R, Allain AS, ElAli A. Non-classical monocytes promote neurovascular repair in cerebral small vessel disease associated with microinfarctions via CX3CR1. J Cereb Blood Flow Metab. 2023;43:1873–90. 37340860 10.1177/0271678X231183742 PMC10676133

[87] Geissmann F, Jung S, Littman DR. Blood monocytes consist of two principal subsets with distinct migratory properties. Immunity. 2003;19:71–82. 12871640 10.1016/s1074-7613(03)00174-2

[88] Privratsky JR, Ide S, Chen Y, Kitai H, Ren J, Fradin H, et al. A macrophage-endothelial immunoregulatory axis ameliorates septic acute kidney injury. Kidney Int. 2023;103:514–28. 36334787 10.1016/j.kint.2022.10.008 PMC9974788

[89] Ancuta P, Wang J, Gabuzda D. CD16^+^ monocytes produce IL-6, CCL2, and matrix metalloproteinase-9 upon interaction with CX3CL1-expressing endothelial cells. J Leukoc Biol. 2006;80:1156–64. 17056766 10.1189/jlb.0206125

[90] Rennert K, Heisig K, Groeger M, Wallert M, Funke H, Lorkowski S, et al. Recruitment of CD16(+) monocytes to endothelial cells in response to LPS-treatment and concomitant TNF release is regulated by CX3CR1 and interfered by soluble fractalkine. Cytokine. 2016;83:41–52. 27031442 10.1016/j.cyto.2016.03.017

[91] Finsterbusch M, Hall P, Li A, Devi S, Westhorpe CL, Kitching AR, et al. Patrolling monocytes promote intravascular neutrophil activation and glomerular injury in the acutely inflamed glomerulus. Proc Natl Acad Sci USA. 2016;113:E5172–81. 27528685 10.1073/pnas.1606253113 PMC5024581

[92] Chen F, Guo S, Li Y, Lu Y, Liu L, Chen S, et al. Fusobacterium nucleatum-driven CX3CR1^+^ PD-L1^+^ phagocytes route to tumor tissues and reshape tumor microenvironment. Gut Microbes. 2024;17:2442037. 10.1080/19490976.2024.2442037 PMC12931713 39710592

[93] Gubin MM, Esaulova E, Ward JP, Malkova ON, Runci D, Wong P, et al. High-dimensional analysis delineates myeloid and lymphoid compartment remodeling during successful immune-checkpoint cancer therapy. Cell. 2018;175:1014–30.e19. 30343900 10.1016/j.cell.2018.09.030 PMC6501221

[94] Lachota M, Zielniok K, Palacios D, Kanaya M, Penna L, Hoel HJ, et al. Mapping the chemotactic landscape in NK cells reveals subset-specific synergistic migratory responses to dual chemokine receptor ligation. EBioMedicine. 2023;96:104811. 37741009 10.1016/j.ebiom.2023.104811 PMC10520535

[95] Russick J, Joubert PE, Gillard-Bocquet M, Torset C, Meylan M, Petitprez F, et al. Natural killer cells in the human lung tumor microenvironment display immune inhibitory functions. J Immunother Cancer. 2020;8:e001054. 10.1136/jitc-2020-001054 PMC7570244 33067317

[96] Singh S, Urs AB, Kumar P. Expression and analysis of CX3CL1 chemokine and CD57^+^ lymphocytes in oral squamous cell carcinoma and their correlation with clinicopathologic features. J Cancer Res Ther. 2024;20:770–5. 39023581 10.4103/jcrt.jcrt_79_22

[97] Zhang X, Lou Y, Zheng S, Chang X. HCC-derived CX3CL1 affects hepatocellular carcinoma prognosis and CX3CR1^+^ MDSC infiltration. Eur J Med Res. 2025;30:153. 40051011 10.1186/s40001-025-02410-z PMC11884201

[98] Pozniak J, Roda N, Landeloos E, Antoranz A, Van Herck Y, De Visscher A, et al. Cytotoxic NK cells impede response to checkpoint immunotherapy in melanoma with an immune-excluded phenotype. Cancer Discov. 2025;15:819–34. 10.1158/2159-8290.CD-24-1208 PMC12409281 40530504

[99] Mei N, Su H, Gong S, Du H, Zhang X, Wang L, et al. High CX3CR1 expression predicts poor prognosis in paediatric acute myeloid leukaemia undergoing hyperleukocytosis. Int J Lab Hematol. 2023;45:53–63. 36064206 10.1111/ijlh.13963 PMC10087374

[100] Xu F, Gao Y, Li T, Jiang T, Wu X, Yu Z, et al. Single-cell sequencing reveals the heterogeneity of hepatic natural killer cells and identifies the cytotoxic natural killer subset in schistosomiasis mice. Int J Mol Sci. 2025;26:3211. 10.3390/ijms26073211 PMC11989782 40244063

[101] Mudd JC, Panigrahi S, Kyi B, Moon SH, Manion MM, Younes SA, et al. Inflammatory function of CX3CR1^+^ CD8^+^ T cells in treated HIV infection is modulated by platelet interactions. J Infect Dis. 2016;214:1808–16. 27703039 10.1093/infdis/jiw463 PMC5142088

[102] Zwijnenburg AJ, Pokharel J, Varnaite R, Zheng W, Hoffer E, Shryki I, et al. Graded expression of the chemokine receptor CX3CR1 marks differentiation states of human and murine T cells and enables cross-species interpretation. Immunity. 2023;56:1955–74.e10. 37490909 10.1016/j.immuni.2023.06.025

[103] Tran KA, Pernet E, Sadeghi M, Downey J, Chronopoulos J, Lapshina E, et al. BCG immunization induces CX3CR1(hi) effector memory T cells to provide cross-protection via IFN-gamma-mediated trained immunity. Nat Immunol. 2024;25:418–31. 38225437 10.1038/s41590-023-01739-z

[104] Akiyama M, Wakasugi S, Yoshimoto K, Saito K, Ishigaki S, Inukai R, et al. CX3CR1(+) age-associated CD4(+) T cells contribute to synovial inflammation in late-onset rheumatoid arthritis. Inflamm Regen. 2025;45:4. 39910629 10.1186/s41232-025-00367-4 PMC11800492

[105] Loredan DG, Devlin JC, Khanna KM, Loke P. Recruitment and maintenance of CX3CR1^+^ CD4^+^ T cells during helminth infection. J Immunol. 2024;212:632–44. 38180236 10.4049/jimmunol.2300451 PMC10954162

[106] Gerlach C, Moseman EA, Loughhead SM, Alvarez D, Zwijnenburg AJ, Waanders L, et al. The chemokine receptor CX3CR1 defines three antigen-experienced CD8 T Cell subsets with distinct roles in immune surveillance and homeostasis. Immunity. 2016;45:1270–84. 27939671 10.1016/j.immuni.2016.10.018 PMC5177508

[107] Ishigaki H, Yamauchi T, Long MD, Hoki T, Yamamoto Y, Oba T, et al. Generation, transcriptomic states, and clinical relevance of CX3CR1^+^ CD8 T cells in melanoma. Cancer Res Commun. 2024;4:1802–14. 38881188 10.1158/2767-9764.CRC-24-0199 PMC11267618

[108] Rica R, Waldherr M, Miyakoda E, Kutschat AP, Schulein M, Zhang J, et al. HDAC1 controls the generation and maintenance of effector-like CD8^+^ T cells during chronic viral infection. J Exp Med. 2025;222:e20240829. 10.1084/jem.20240829 PMC12135962 40464916

[109] Abdelfatah E, Long MD, Kajihara R, Oba T, Yamauchi T, Chen H, et al. Predictive and prognostic implications of circulating CX3CR1^+^ CD8^+^ T Cells in non-small cell lung cancer patients treated with chemo-immunotherapy. Cancer Res Commun. 2023;3:510–20. 37009132 10.1158/2767-9764.CRC-22-0383 PMC10060186

[110] Cassidy BR, Zhang M, Sonntag WE, Drevets DA. Neuroinvasive Listeria monocytogenes infection triggers accumulation of brain CD8^+^ tissue-resident memory T cells in a miR-155-dependent fashion. J Neuroinflamm. 2020;17:259. 10.1186/s12974-020-01929-8 PMC7466815 32878636

[111] Yang H, Yang J, Park N, Hwang DS, Park SY, Kim S, et al. Adoptive transfer of CX3CR1-transduced tregs homing to the forebrain in lipopolysaccharide-induced neuroinflammation and 3xTg Alzheimer’s disease models. Int J Mol Sci. 2024;25:13682. 10.3390/ijms252413682 PMC11727661 39769442

[112] Hiddingh S, Pandit A, Verhagen F, Rijken R, Servaas NH, Wichers R, et al. Transcriptome network analysis implicates CX3CR1-positive type 3 dendritic cells in non-infectious uveitis. Elife. 2023;12:e74913. 10.7554/eLife.74913 PMC10185339 37042831

[113] Zhuang J, Li X, Yao J, Sun X, Liu J, Nie H, et al. Single-cell RNA sequencing reveals the local cell landscape in mouse epididymal initial segment during aging. Immun Ageing. 2023;20:21. 37170325 10.1186/s12979-023-00345-9 PMC10173474

[114] Mohapatra ANB, Howard Z, Ernst JD. CCR2 recruits monocytes to the lung, while CX3CR1 modulates positioning of CD11cpos cells in the lymph node during pulmonary tuberculosis. mBio. 2025;16:e0123725. 10.1128/mbio.01237-25 PMC12239560 40497732

[115] Zheng LY, Duan Y, He PY, Wu MY, Wei ST, Du XH, et al. Dysregulated dendritic cells in sepsis: functional impairment and regulated cell death. Cell Mol Biol Lett. 2024;29:81. 38816685 10.1186/s11658-024-00602-9 PMC11140885

[116] Nyasha MR, Chen W, Wang H, Yaoita F, Aoki M, Nagatomi R, et al. Effects of CX3CR1 and CXCR2 antagonists on running-dependent intramuscular neutrophil recruitments and myokine upregulation. Am J Physiol Endocrinol Metab. 2023;324:E375–E89. 36856190 10.1152/ajpendo.00196.2022

[117] Liu H, Wang P, Yin J, Yang P, Shi J, Li A, et al. High expression of CX3CL1/CX3CR1 at the mother-fetus interface of preeclampsia inhibits trophoblast invasion and migration. Placenta. 2024;156:30–7. 39236525 10.1016/j.placenta.2024.08.008

[118] Shih HL, Cheng KH, Chen CH, Chang JY, Hsu KS. Upregulation of cathepsin s expression contributes to neuronal damage following kainic acid-induced status epilepticus. J Neurochem. 2025;169:e70037. 40042007 10.1111/jnc.70037

[119] Murai N, Mitalipova M, Jaenisch R. Functional analysis of CX3CR1 in human induced pluripotent stem (iPS) cell-derived microglia-like cells. Eur J Neurosci. 2020;52:3667–78. 32579729 10.1111/ejn.14879 PMC8168698

[120] Huang J, Liu H, Liu Z, Wang Z, Xu H, Li Z, et al. Inhibition of aortic CX3CR1^+^ macrophages mitigates thoracic aortic aneurysm progression in Marfan syndrome in mice. J Clin Invest. 2025;135:e178198. 10.1172/JCI178198 PMC11735105 39817456

[121] Cao Q, Wang Y, Chen J, Wang R, Chen T, Gloss B, et al. Targeting inflammation with chimeric antigen receptor macrophages using a signal switch. Nat Biomed Eng. 2025;9:1502–16. 10.1038/s41551-025-01387-8 PMC12443588 40335685

[122] Xu J, Liu L, Parone P, Xie W, Sun C, Chen Z, et al. In-vivo B-cell maturation antigen CAR T-cell therapy for relapsed or refractory multiple myeloma. Lancet. 2025;10500:228–31. 10.1016/S0140-6736(25)01030-X 40617243

[123] Hunter TLBY, Zhang Y, Matsuda D, Riener R, Wang A, Li JJ, Soldevila F, Chu DSH, Nguyen DP, Yong QC, Ross B, Nguyen M, Vestal J, Roberts S, Galvan D, Vega JB, Jhung D, Butcher M, Nguyen J, Zhang S, Fernandez C, Chen J, Herrera C, Kuo Y, Pica EM, Mondal G, Mammen AL, Scholler J, Tanis SP, Sievers SA, Frantz AM, Adams GB, Shawver L, Farzaneh-Far R, Rosenzweig M, Karmali PP, Bot AI, June CH, Aghajanian H. In vivo CAR T cell generation to treat cancer and autoimmune disease. Science. 2025;388:1311–7. 40536974 10.1126/science.ads8473

[124] Ho C-Y, Lin Y-T, Chen H-H, Ho W-Y, Sun G-C, Hsiao M, et al. CX3CR1-microglia mediates neuroinflammation and blood pressure regulation in the nucleus tractus solitarii of fructose-induced hypertensive rats. J Neuroinflamm. 2020;17:185. 10.1186/s12974-020-01857-7 PMC7291459 32532282

[125] Ridderstad Wollberg A, Ericsson-Dahlstrand A, Jureus A, Ekerot P, Simon S, Nilsson M, et al. Pharmacological inhibition of the chemokine receptor CX3CR1 attenuates disease in a chronic-relapsing rat model for multiple sclerosis. Proc Natl Acad Sci USA. 2014;111:5409–14. 24706865 10.1073/pnas.1316510111 PMC3986185

[126] Xie J, Barbolina MV. Dual targeting of CX(3)CR1 and PARP in models of high-grade serous ovarian carcinoma. Cancers. 2024;16:3728. 10.3390/cancers16223728 PMC11591600 39594684

[127] Zhang C, Wang X, Liu G, Ren H, Liu J, Jiang Z, et al. CRISPR/Cas9 and chlorophyll coordination micelles for cancer treatment by genome editing and photodynamic therapy. Small. 2023;19:e2206981. 36693779 10.1002/smll.202206981

[128] Tamura R, Yo M, Toda M. Gene therapy and genome-editing for schwannoma in NF2-related schwannomatosis: current understanding and future directions. J Neurooncol. 2025;173:263–73. 10.1007/s11060-025-04995-1 40055258

[129] Israr J, Kumar A. Current progress in CRISPR-Cas systems for rare diseases. Prog Mol Biol Transl Sci. 2025;210:163–203. 39824580 10.1016/bs.pmbts.2024.07.019

[130] Fu J, Cai W, Pan S, Chen L, Fang X, Shang Y, et al. Developments and trends of nanotechnology application in sepsis: a comprehensive review based on knowledge visualization analysis. ACS Nano. 2024;18:7711–38. 38427687 10.1021/acsnano.3c10458

[131] Lin L, Liu H, Zhang D, Du L, Zhang H. Nanolevel immunomodulators in sepsis: novel roles, current perspectives, and future directions. Int J Nanomed. 2024;19:12529–56. 10.2147/IJN.S496456 PMC11600945 39606559

[132] Li YR, Dunn ZS, Yu Y, Li M, Wang P, Yang L. Advancing cell-based cancer immunotherapy through stem cell engineering. Cell Stem Cell. 2023;30:592–610. 36948187 10.1016/j.stem.2023.02.009 PMC10164150

